# Transcriptional changes in chick wing bud polarization induced by retinoic acid

**DOI:** 10.1002/dvdy.24543

**Published:** 2017-07-24

**Authors:** Joseph Pickering, Neha Wali, Matthew Towers

**Affiliations:** ^1^ Bateson Centre Department of Biomedical Science, University of Sheffield Sheffield United Kingdom; ^2^ Sanger Institute, Wellcome Genome Campus Cambridge United Kingdom

**Keywords:** retinoic acid, chick wing, limb, digits, Shh, transcriptomics, Hoxd

## Abstract

**Background**: Retinoic acid is implicated in the induction of the gene encoding Sonic hedgehog (Shh) that specifies anteroposterior positional values and promotes growth of the developing limb bud. However, because retinoic acid is involved in limb initiation, it has been difficult to determine if it could have additional roles in anteroposterior patterning. To investigate this, we implanted retinoic acid–soaked beads to the anterior margin of the chick wing bud and performed microarray analyses prior to onset of *Shh* expression. **Results:** Retinoic acid up‐regulates expression of *Hoxd11‐13* that encode transcription factors implicated in inducing *Shh* transcription and that are involved in digit development. In our assay, retinoic acid induces *Shh* transcription and, consequently, a new pattern of digits at a much later stage than anticipated. Retinoic acid represses many anteriorly expressed genes, including *Bmp4, Lhx9, Msx2*, and *Alx4*. We provide evidence that retinoic acid influences transcription via induction of *dHAND* and inhibition of *Gli3* to establish a new anteroposterior pre‐pattern. We show that transient exposure to retinoic acid can suppress distal development and expedite cells to transcriptionally respond to Shh. **Conclusions:** Our findings reveal how retinoic acid and Shh signaling could cooperate in anteroposterior patterning of the limb. *Developmental Dynamics 246:682–690, 2017*. © 2017 Wiley Periodicals, Inc.

## Key Findings

- Retinoic acid‐soaked beads implanted into the anterior margin of an early chick wing bud can induce a new pattern of digits with only a 12 h requirement of Shh expression.
- Retinoic acid induces dHAND and inhibits Gli3 expression to establish a new antero‐posterior pre‐pattern.
- Retinoic acid induces expression of 5'Hoxd genes independently of Shh signalling.
- Retinoic acid represses many anteriorly‐expressed genes.
- Retinoic acid expedites the response of cells to Shh signalling.

## Introduction

One of the earliest discovered effects of retinoic acid signaling on limb development was in influencing patterning along the anteroposterior axis (thumb to little finger) of the chick wing (Tickle et al., 1982). Beads soaked in retinoic acid and then implanted to the anterior margin of the early chick wing bud elicit mirror‐image duplications of the pattern of three digits, 1, 2 and 3, to produce patterns such as 3, 2, 1, 1, 2, and 3 (Tickle et al., 1982; Tickle et al., 1985). Such duplicated digit patterns are similar to those obtained when a specialized group of posterior mesenchyme cells—known as the polarizing region—are grafted to the anterior margins of host wing buds (Saunders and Gasseling, 1968). Based on these observations, it was suggested that retinoic acid could be the sought after morphogen produced by the polarizing region that specifies cells with positional values across the anteroposterior axis in a concentration‐dependent manner (Wolpert, 1969; Tickle et al., 1975). Thus, low concentrations of retinoic acid specify positional values appropriate to specify the anterior digit 1, increasing levels, the middle digit 2, and then the posterior digit 3 (Tickle et al., 1982; Tickle et al., 1985). In support of retinoic acid being the morphogen, it was demonstrated to be present in chick wing buds and distributed in a graded manner with the highest levels posteriorly (Thaller and Eichele, 1987). However, it was later shown that retinoic acid induces a new polarizing region (Noji et al., 1991; Wanek et al., 1991) and that its effects on specifying a new pattern of digits are mediated by the secreted peptide encoded by the *Sonic hedgehog* (*Shh*) gene (Riddle et al., 1993; Helms et al., 1994). It is not clear if retinoic acid is involved in the initiation of *Shh* expression in normal limb development. There is, however, evidence from both mouse and chick studies that retinoic acid is required for forelimb initiation (Helms et al., 1996; Stratford et al., 1996; Niederreither et al., 2002). It is also possible that retinoic acid is involved in the establishment and/or maintenance of the anteroposterior polarity of the limb, which is marked by anterior expression of *Gli3* and posterior expression of *dHAND* (reviewed in Tickle, 2015). Another proposed role for retinoic acid signaling is in specifying the positional values of the most proximal part of the forelimb (the humerus) by regulating expression of genes encoding Meis1/2 transcription factors (Capdevila et al., 1999; Mercader et al., 1999; Mercader et al., 2000; Rosello‐Diez et al., 2011), although this role is controversial (Cunningham et al., 2013).

In this study, we have further examined how retinoic acid signaling could influence anteroposterior patterning by implanting retinoic acid–soaked beads to the anterior margin of the chick wing bud under conditions in which ectopic *Shh* is not induced until late bud stages. We then carried out microarray analysis to identify transcriptional changes that occur prior to the onset of *Shh* expression. We confirm that retinoic acid signaling induces the expression of *dHAND* and *5′Hoxd* genes, which are normally posteriorly expressed, and encode transcription factors involved in the transcriptional initiation of *Shh* (Charite et al., 2000; Zakany et al., 2004). Additionally, we reveal that retinoic acid signaling inhibits many anteriorly expressed genes, including *Bmp4* and *Lhx9*, and we propose that this occurs because *Gli3* transcription is repressed. We provide evidence that retinoic acid allows cells to express *Shh* and then rapidly respond to Shh signaling.

## Results

### Retinoic Acid Can Induce *Shh* Expression at a Later Stage than Anticipated

**Figure 1:**
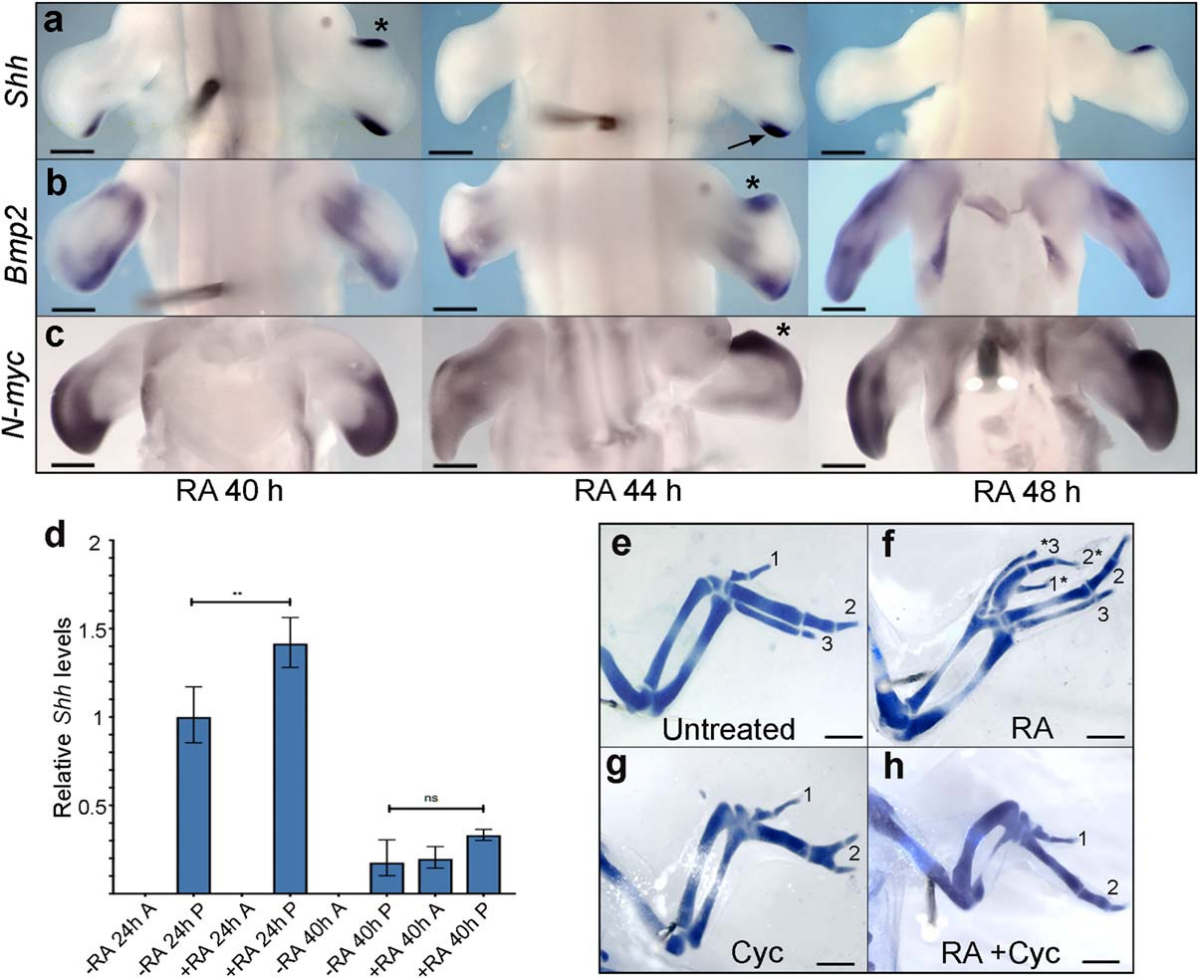
Effects of retinoic acid on gene expression and digit pattern. Application of retinoic acid (RA)–soaked beads to the anterior margin of HH20 wing buds induces *Shh* expression after approximately 40 hr (**A**, expression persists until about 48 h), *Bmp2* after 44 hr (**B**), and *N‐myc* after 44 hr (**C**). **D**: Quantitative RT‐PCR shows that retinoic acid does not induce *Shh* after 24 hr but induces *Shh* after 40 hr (A, anterior; P, posterior; + RA, retinoic acid–treated wings; ‐RA, untreated wings). Up‐regulation of *Shh* expression in + RA 24h P compared to ‐RA 24h A is significant (unpaired *t*‐test; ** *P* = 0.0067), but not in +RA 40h P compared to ‐RA 40h P (unpaired *t*‐test; *P* = 0.0907). Differences in *Shh* expression are relative to untreated posterior tissue at HH24. Untreated chick wing (**E**) and application of retinoic acid beads at HH20 results in digit duplications (**F;** see Supplementary Table 2). Application of cyclopamine (cyc) to HH20 embryos often results in loss of digit 3 (**G**). Treatment with retinoic acid beads and cyclopamine together at HH20 produces similar patterns (**H**) to those obtained with cyclopamine only. Scale bars A–C = 500 μm. Scale bars E–H = 1 mm. Error bars indicate standard error.

To gain insights into the effects of retinoic acid on anteroposterior patterning, we implanted AG1‐X2 beads soaked in retinoic acid to the anterior margins of stage HH20 wing buds. Previous studies have shown that 0.01‐µg/µl^‐1^ to 1‐µg/µl^‐1^ concentrations of retinoic acid loaded on beads 200–250 μm in diameter induced *Shh* expression after 22–24 hr at Hamburger Hamilton stage 24 (HH24) (Helms et al., 1994). However, we discovered that a 5‐μg/μl^‐1^ concentration of retinoic acid loaded on 150‐μm beads did not induce *Shh* expression until approximately 40 hr at HH26 (n = 6/7, Fig. 1A; Supplementary Table 1, n = 2/12 at 36 hr). In such wing buds, *Shh* expression persisted at high levels for around 8 hr until HH27 and terminated after approximately 12 hr (Fig. 1A, note arrow showing retinoic acid treatment extending duration of endogenous *Shh* expression; see Chinnaiya et al., 2014). Quantitative RT‐PCR analyses confirmed that *Shh* expression could not be detected in the anterior regions of treated wing buds at 24 hr, but could be detected at 40 hr (Fig. 1D). In addition, the extended duration of endogenous *Shh* expression detected by in situ hybridization (Fig. 1A) was also detected by quantitative RT‐PCR, as were increased levels of endogenous *Shh* expression at 24 hr and at 40 hr (Fig. 1D; compare contralateral buds for increased levels at 40 hr; Fig. 1A). Thus, even when retinoic acid beads are implanted anteriorly, there is a delay in the intrinsically timed expression of endogenous *Shh*, with increased levels being observed at later stages than usual (Chinnaiya et al., 2014).

Four hours after the anterior induction of *Shh*, the expression of known downstream targets of Shh signaling was observed, including *Bmp2* (n = 3/3, Fig. 1B) and *N‐myc* (n = 3/3, Fig. 1C). The products of these genes are implicated in anteroposterior specification and proliferation/growth, respectively, thus providing evidence that these two functions are integrated by Shh signaling, as previously proposed (Drossopoulou et al., 2000; Towers et al., 2008). To examine if a 5‐μg/μl^‐1^ concentration of retinoic acid applied on 150‐μm beads at HH20 is sufficient to induce the formation of additional digits, we analyzed skeletal development at day 10, and this revealed that wings frequently formed with an additional digit 3 (n = 23/26, Fig. 1F; Supplementary Table 2, note untreated wing; Fig. 1E). In addition, polarizing activity was weaker in experiments in which a 1‐μg/μl^‐1^ concentration of retinoic acid was applied on 200‐μm beads (additional digit 3, n = 3/11, Supplementary Table 2). Therefore, the polarizing activity produced by high concentrations of retinoic acid is equivalent to that of other studies in which lower concentrations of retinoic acid were loaded onto larger beads (Tickle et al., 1982; Tickle et al., 1985; Helms et al., 1994). The differences in the results presented here compared with those of earlier studies could also be caused by retinoic acid batch and the general variability inherent to these kinds of experiments. The important factor to consider is the amount of active retinoic acid in the tissue, which is difficult to determine (Tickle et al., 1985).

Our finding that ectopic *Shh* expression at HH26 correlates with the formation of additional digits is surprising, as Shh specifies anteroposterior positional values between HH18/19 and HH21 in normal chick wing development (Towers et al., 2011; Pickering and Towers, 2016), and polarizing region grafts made to the anterior margin of chick wing buds after HH23 fail to duplicate the pattern of digits (Summerbell, 1974). Therefore, to determine whether Shh signaling is inducing a new pattern of digits after HH26 (Fig. 1A), we treated embryos at HH20 with both retinoic acid (5 μg/μl^‐1^ on 150‐μm beads, conditions used throughout rest of article) and cyclopamine, an inhibitor of Shh signaling at the level of Smoothened. Consistent with previous studies (Scherz et al., 2008; Towers et al., 2011; Pickering and Towers, 2016), treatment of HH20 embryos with cyclopamine resulted in absence of digit 3 in the majority of cases (n = 5/8, Fig. 1G, Supplementary Table 2). Similarly, treatment of embryos with both cyclopamine and retinoic acid at HH20 also resulted in absence of digit 3; in addition, no additional anterior digits were observed (n = 4/6, Fig. 1H; Supplementary Table 2). These results show that retinoic acid can induce *Shh* at a later stage than anticipated.

### Microarray Analyses of Retinoic Acid–treated Chick Wing Buds

Since high concentrations of retinoic acid supplied on beads can induce *Shh* expression at later stages of development than previously reported, this facilitates investigation into the earlier effects of retinoic acid signaling on anteroposterior patterning. To achieve this, we implanted retinoic acid–soaked beads to the anterior margins of HH20 wing buds, and after 24 hr at HH24 (12–16 hr before anterior *Shh* induction, Fig. 1A), we dissected anterior thirds from which we extracted RNA that was then used to probe chicken Affymetrix gene arrays (see Experimental Procedures; note that wing buds that showed perturbed outgrowth were excluded from the analysis). To assess the effectiveness of retinoic acid treatment in this experiment, several of the manipulated embryos were left to develop until day 10, and most wings formed an anterior digit 3 (n = 14/22, Supplementary Table 3).

**Figure 2:**
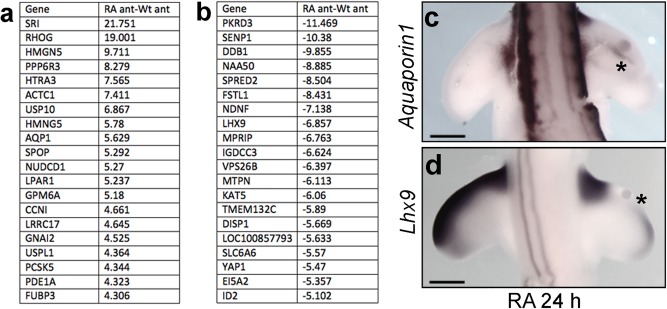
Microarray analyses of retinoic acid‐treated wing buds. **A**: Top 20 genes up‐regulated by retinoic acid–soaked beads implanted to the anterior margins of HH20 wing buds after 24 hr (note adjusted fold‐change is shown; see Supplementary Dataset 1). **B**: Top 20 genes down‐regulated by retinoic acid–soaked beads implanted to the anterior margins of HH20 wing buds after 24 hr. Expression of *Aquaporin1* (*Aqp1*) is observed adjacent to the retinoic acid–soaked bead after 24 hr (asterisk in **C**), and the anterior‐distal domain of *Lhx9* expression is down‐regulated after 24 hr (asterisk in **D**). Scale bars = 500 μm.

In total, 1288 features on the array were differentially expressed (adjusted *P* < 0.01) by > two‐fold in the anterior of retinoic acid–treated wing buds compared with the equivalent anterior region of HH24 control wing buds (Supplementary Dataset 1). Of these, 292 were increased in expression and 996 were decreased in expression (Fig. 2A,B, top 20 genes; Supplementary Dataset 1). These microarray data reveal that retinoic acid signaling alters the expression of many genes prior to its transcriptional induction of *Shh*.

We confirmed that the results of the microarray experiment represent changes in gene expression levels by RNA in situ hybridization. *Aquaporin1* (*Aqp1*) encodes a protein involved in ion channel communication (Benga, 2012), and although not normally expressed in the wing bud, transcripts were induced close to retinoic‐soaked beads toward the center of the bud after 24 hr (n = 4/5, Fig. 2C). The expression pattern of this gene suggests that it is not involved in establishing the polarizing region, or in responding to Shh signaling by the polarizing region, which forms distal to the retinoic acid–loaded bead and in contact with the apical ectodermal ridge, thus ensuring cross talk between this structure and the underlying mesenchyme. In addition, expression of *Lhx9*, which encodes a LIM homeodomain transcription factor implicated in limb patterning (Tzchori et al., 2009), was repressed by retinoic acid treatment in the distal‐anterior region of the bud after 24 hr (n = 6/6, Fig. 2D).

### Retinoic Acid Induces *5′Hoxd* Expression Independent of Shh

Many of the genes indicated by the microarray data as being responsive to retinoic acid signaling have not previously been associated with limb development, and thus, like *Aqp1*, may not even be expressed in the limb bud. Therefore, to focus on genes expressed in the developing limb and that could be involved in anteroposterior patterning in response to retinoic acid, we performed clustering analyses on pairwise comparisons between the anterior thirds of retinoic acid–treated and –untreated HH24 wing buds, between the anterior and posterior thirds of HH24 wings buds (Bangs et al., 2010) and between the anterior thirds of *talpid* ^*3*^ mutant and wild‐type HH24 wing buds (Bangs et al., 2010; see also Supplementary Dataset 1). The wild‐type anterior to wild‐type posterior comparison was used to enrich for genes that could be involved in anteroposterior patterning. The *talpid^3^* anterior to wild‐type anterior comparison was used to identify those genes that are downstream of the transcriptional repressor Gli3 and that reflect anterior‐to‐posterior re‐specification. The chicken *talpid* ^*3*^ mutant is defective in the processing of Gli3 to the repressor form (Gli3R). In normal development, Shh signaling prevents this processing event, allowing the posterior expression of genes such as *Hoxd13* (Davey et al., 2006; Bangs et al., 2010). In *talpid* ^*3*^‐mutant wing buds, Gli3 function is lost and many putative targets of Shh signaling, which are normally repressed by Gli3, such as *Hoxd13*, become ectopically expressed in the anterior part of the wing bud; in addition, expression of some anteriorly expressed genes is also lost, suggesting that Gli3 normally represses a transcriptional inhibitor of these genes (Bangs et al., 2010).

**Figure 3:**
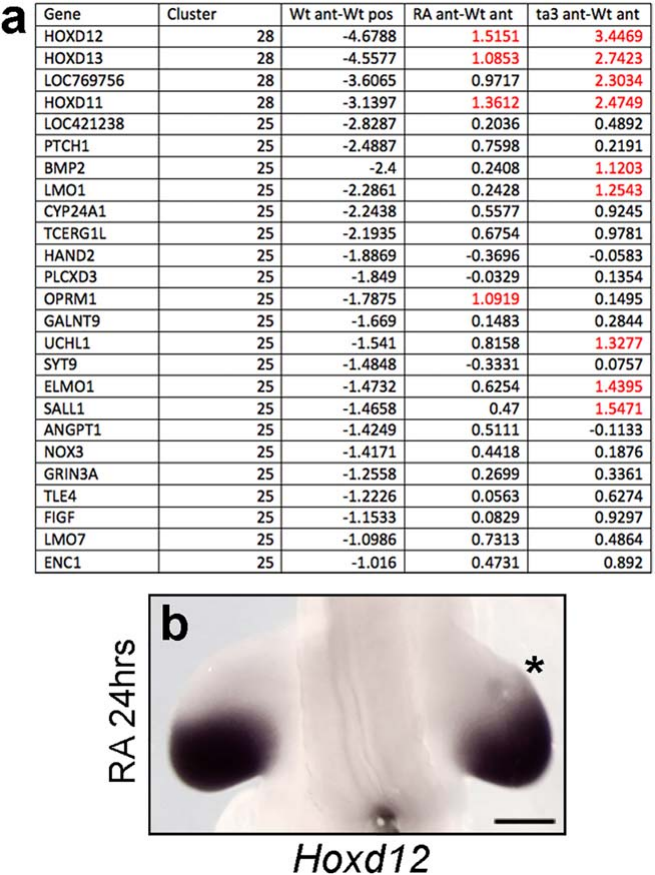
Posterior genes up‐regulated anteriorly both by retinoic acid and in *talpid* ^*3*^. **A**: Red indicates genes that have a > two‐fold change (note adjusted log‐fold values are shown). Expression of *Hoxd12* is up‐regulated adjacent to the retinoic acid–soaked bead after 24 hr (asterisk in **B**). Scale bar = 500 μm.

Twenty‐nine gene clusters were identified from the set of 1324 unique genes that were differentially expressed ( > two‐fold, adjusted *P* < 0.0001) across at least one of the three pairwise comparisons, Supplementary Datasets 2 and 3; see Experimental Procedures). To find targets of retinoic acid signaling that could be involved in anteroposterior patterning, we focused on those clusters that contained genes expressed at higher levels in the posterior part of the wing bud. In total, 25 unique genes that were found in two clusters exhibited > two‐fold higher expression in the posterior part of the wing bud compared with the anterior (Fig. 3A, shown as log2‐fold changes; i.e., 1 is a two‐fold change, 2 is a four‐fold change). Of these, only four were induced > two‐fold in the anterior region of the wing bud in response to retinoic acid signaling, and notably, three of these encode the most‐5′ genes of the *Hoxd* gene cluster (*d11, d12*, and *d13*), the other gene being *Opioid Receptor Mu 1* (*OPRM1*) (Figs. 3A,B, showing *Hoxd12* expression, n = 3/4). The three *Hoxd* genes are expressed in the anterior regions of *talpid* ^*3*^ wing buds, showing that Gli3 normally represses them (Bangs et al., 2010). Several genes were identified that, although not responsive to retinoic acid signaling, are repressed by Gli3 in the anterior of the wing bud; these include *Bmp2* and *Sall1*, which are potentially involved in the specification of anteroposterior positional values (Drossopoulou et al., 2000; Welten et al., 2011). Therefore, these data reveal that retinoic acid signaling significantly up‐regulates the expression of four genes in a Shh‐independent manner, which are potentially involved in anteroposterior patterning; these include three *5′Hoxd* genes that are normally repressed anteriorly by Gli3.

### Retinoic Acid Represses Many Anterior Genes

**Figure 4:**
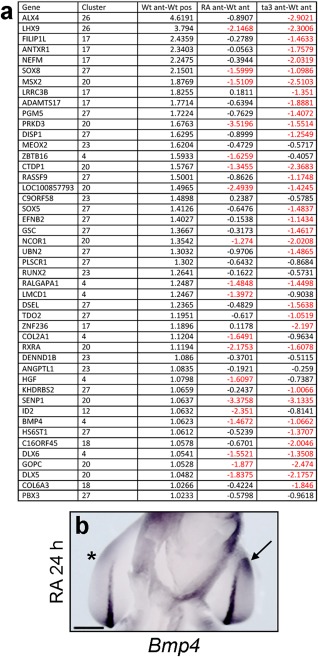
Anterior genes down‐regulated anteriorly both by retinoic acid and in *talpid* ^*3*^. **A**: Red indicates genes that have a > two‐fold change (note adjusted log‐fold values are shown. Expression of *Bmp4* is down‐regulated adjacent to the retinoic acid–soaked bead after 24 hr (asterisk in **B**. Compare with expression in contralateral wing bud in equivalent position [arrow]; note ventral view of embryo is shown). Scale bar = 500 μm.

To find genes potentially involved in anteroposterior patterning that retinoic acid signaling represses in the anterior part of the wing bud, we analyzed clusters containing genes that are expressed > two‐fold higher in the anterior regions of normal wing buds compared with the posterior. In total, 46 genes were found in eight different clusters, including *Alx4, Bmp4*, and *Msx2* (Fig. 4A; Supplementary Datasets 2 and 3). Further analysis of the data revealed that retinoic acid signaling represses 19 of these genes (41%), including *Bmp4* (n = 3/3, Fig. 4B). It is notable that both retinoic acid signaling and loss of Gli3 function in *talpid* ^*3*^ embryos results in the down‐regulation of many of the same anteriorly expressed genes, including *Lhx9* and *Bmp4* (Fig. 4A). Thus, 14 out of 19 (74%) anteriorly expressed genes repressed by retinoic acid signaling are also repressed in *talpid* ^*3*^ (Fig. 4A). These data show that many of the genes that are repressed by retinoic acid are also those that depend on Gli3 function for their expression.

### Retinoic Acid Inhibits *Gli3* and Induces *dHAND*

**Figure 5:**
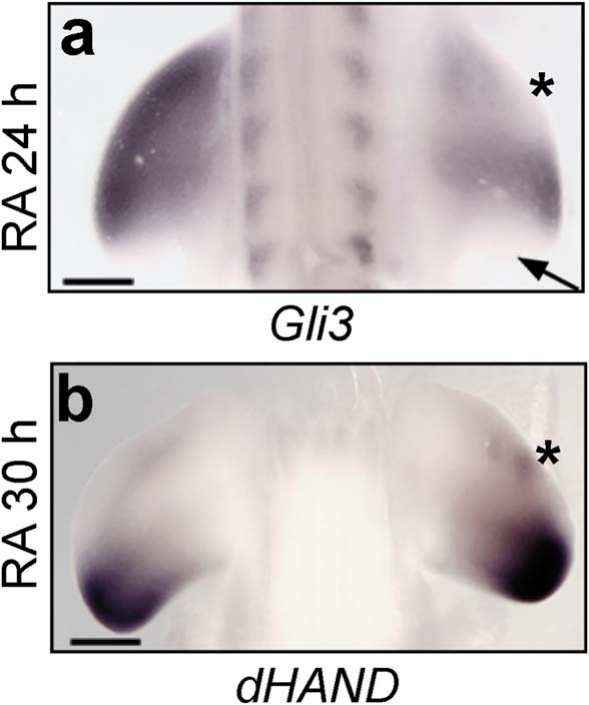
Retinoic acid affects the expression of pre‐patterning genes. Application of retinoic acid–soaked beads to the anterior margins of HH20 wing buds down‐regulates *Gli3* after 24 hr (asterisk in **A;** note normal absence of expression posteriorly [arrow]). Ectopic *dHAND* is observed adjacent to retinoic acid–soaked beads after 30 hr (asterisk in **B**). Scale bars = 500 μm.

How can we explain the overlap in genes that are transcriptionally repressed either by loss of Gli3 function in *talpid* ^*3*^ or by retinoic acid signaling? One possibility is that retinoic acid affects *Gli3* transcription. Indeed, 24 hr after retinoic acid–soaked beads were grafted to the anterior regions of HH20 chick wing buds, *Gli3* expression was reduced (n = 6/6, asterisk in Fig. 5A; note reduced expression of *Gli3* in posterior regions in response to endogenous Shh signaling [arrow]). Although only a slight decrease in *Gli3* expression was indicated by the microarray data (adjusted 1.2‐fold), this is representative of the anterior third of the wing bud, which includes the region of the wing bud where *Gli3* is expressed. Gli3 and dHAND mutually antagonize each other's expression to provide the early limb bud with an inherent anterior‐posterior pre‐pattern (te Welscher et al., 2002). In addition, retinoic acid is implicated in regulating *dHAND* expression (Ros et al., 2003), which, in turn, contributes to the transcriptional initiation of *Shh* (Charite et al., 2000). However, although we did not detect changes in *dHAND* expression in the microarray experiment after 24 hr exposure to retinoic acid, ectopic expression could be detected by in situ hybridization after 30 hr (n = 4/4, asterisk in Fig. 5B). Therefore, these findings suggest that retinoic acid can establish reciprocal patterns of *dHAND* and *Gli3* expression.

### Retinoic Acid Allows Cells to Rapidly Respond to Shh Signaling

**Figure 6:**
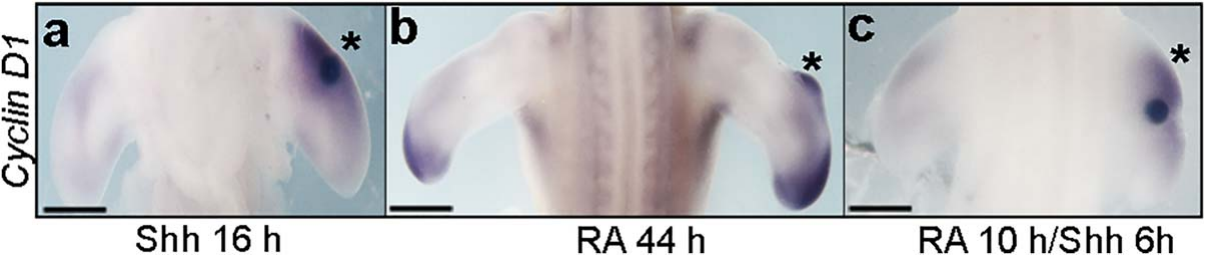
Retinoic acid exposure allows cells to transcriptionally respond to Shh. Application of Shh‐soaked beads to the anterior margins of HH20 wing buds up‐regulates *Cyclin D1* after 16 hr (asterisk in **A**). Application of retinoic acid–soaked beads to the anterior margins of HH20 wing buds up‐regulates *Cyclin D1* after 44 hr (asterisk in **B**). Application of retinoic acid–soaked beads to the anterior margins of HH20 wing buds, removed after 10 hr and then replaced with Shh‐soaked beads, results in up‐regulation of *Cyclin D1* expression after a further 6 hr (asterisk in **C**). Scale bars = 500 μm.

One of the aspects of the retinoic acid treatment protocol used here is that the induction of downstream target genes of Shh signaling, such as *Bmp2*, occurs at a shorter interval after Shh induction than previously reported (Francis‐West et al., 1994; Helms et al., 1994). Furthermore, previous studies have shown that when Shh is applied on beads to the anterior margins of chick wing buds, transcriptional targets such as *Bmp2* and *5′Hoxd* genes require 16–24 hr to be expressed, yet following retinoic acid treatment, are expressed almost coincidently with *Shh* after around 24 hr (Francis‐West et al., 1994; Helms et al., 1994). This suggests that retinoic acid may expedite the response of cells to Shh signaling. To test this possibility, we therefore investigated the effects of retinoic acid on the timing of induction of the Shh target gene, *Cyclin D1* (Towers et al., 2008). When Shh‐soaked beads were implanted to the anterior margins of HH20 chick wing buds, expression of *Cyclin D1* was observed after 16 hr (n = 13/16, Fig. 6A) as previously reported (Towers et al., 2008). In contrast, expression of *Cyclin D1* was observed 44 hr after retinoic acid–soaked beads were implanted to the anterior margin of HH20 wing buds using our protocol (n = 8/9, Fig. 6B), only 4 hr after the induction of *Shh* transcription (Fig. 1A). Therefore, to test whether a short exposure of anterior tissue to retinoic acid can hasten gene expression, we replaced a retinoic acid–soaked bead after 10 hr with another bead soaked in Shh. Indeed, 6 hr after application of the Shh‐soaked bead, expression of *Cyclin D1* could be detected in the anterior mesenchyme (n = 4/6, Fig. 6C), 10 hr sooner than in tissue not previously exposed to retinoic acid (Fig. 6A). These data suggest that retinoic acid signaling can give anterior wing bud mesenchyme competence to transcriptionally respond to Shh signaling.

## Discussion

We have shown that the application of retinoic acid to the anterior margin of the HH20 chick wing bud can induce a new pattern of digits after HH26 in a Shh‐dependent manner, a much later stage than anticipated. By analyzing genes that are differentially expressed across the anteroposterior axis of the wing bud, we have revealed that retinoic acid can establish a new anteroposterior pre‐pattern of gene expression independently of Shh by inducing *dHAND* and repressing *Gli3*. We suggest that these events suppress an anterior developmental program, characterized by the expression of genes including *Bmp4, Lhx9* and *Msx2*, and induce a posterior program, characterized by the expression of genes, such as those of the *5′Hoxd* cluster and *Shh*. We provide evidence, confirming in vitro findings (Ogura et al., 1996), that retinoic acid can facilitate anteroposterior patterning by allowing cells to respond rapidly to Shh signaling.

### Using the Chick Wing Bud to Investigate Roles of Retinoic Acid in Anteroposterior Patterning

The aim of this article was to investigate the effects that retinoic acid can have on anteroposterior patterning, independently of earlier events, such as bud initiation and proximodistal patterning. Therefore, since this involves inducing a new digit pattern by implanting beads soaked in retinoic acid to the anterior margin of the chick wing bud, caution must be exercised when extrapolating our findings onto the possible roles of retinoic acid in normal development. Indeed, our assay demonstrates that *Shh* expression can be induced by retinoic acid at much later stages than previously published (Riddle et al., 1993; Helms et al., 1994), which is likely to be due to the dynamics of the release of higher concentrations by smaller beads used in this study, compared with low concentrations of retinoic acid by larger beads. However, the physiological outcome remains unchanged because the digit duplications obtained in our experiments here are comparable to those reported in earlier studies (Tickle et al., 1982; Tickle et al., 1985; Helms et al., 1994).

### Retinoic Acid in Anteroposterior Pre‐patterning

The anteroposterior polarity of the chick wing bud is specified in the primitive paraxial mesoderm at around HH8/9 (Chaube, 1959, reviewed in Tickle, 2015), some considerable time before wing bud initiation. At around HH8/9, patterns of *Hox* gene expression are being established along the main anteroposterior axis of the embryo (reviewed in Mallo et al., 2010). Elegant genetic experiments in the mouse have recently shown that the products encoded by Hox5 and Hox9 paralogous genes are major determinants of the anterior and posterior forelimb pre‐pattern, respectively: Hox5 paralogues suppress anterior expression of *Shh* (Xu et al., 2013), and Hox9 paralogues promote expression of *dHAND* (Xu and Wellik, 2011), which induces *Shh* expression posteriorly (Charite et al., 2000). dHAND and Gli3 antagonize each other's expression, which causes their posterior and anterior restriction in the early bud (te Welscher et al., 2002). Retinoic acid has previously been implicated in contributing to the regulation of *Hox* gene expression in the main body axis (Reviewed in Deschamps and van Nes, 2005), and therefore it is possible that it is involved in establishing the initial anteroposterior limb pre‐pattern. Indeed, application of retinoic acid to HH10 embryos can interfere with anteroposterior pre‐patterning and polarize chick limbs (Wilde et al., 1987). However, *Hox5* and *Hox9* paralogues are not expressed during wing bud stages and so are unlikely to contribute to the polarization of the anterior part of the bud by retinoic acid. In support of this, our microarray data did not reveal significant changes in *Hox5* or *Hox9* expression following retinoic acid treatment. Our results are therefore more consistent with a possible role for retinoic acid in the continued refinement and/or maintenance of a pre‐pattern at limb bud stages, by contributing to the posterior expression of *Hoxd*/*dHAND* genes, and also to the posterior repression of *Gli3*. In support of this mechanism, retinoic acid is graded across the anteroposterior axis of the early wing bud, with the highest levels posteriorly (Thaller and Eichele, 1987).

### Retinoic Acid in Anteroposterior Patterning

Our finding that retinoic acid can induce *5′Hoxd* and *dHAND* gene expression raises the possibility that this event could contribute to the initiation of *Shh* expression in normal development. We showed that the transient exposure of anterior wing bud cells to retinoic acid expedites the expression of *Cyclin D1* in response to Shh. We speculate that this occurs because *Gli3* expression is repressed. Retinoic acid is present only during early stages of limb bud outgrowth (Mercader et al., 1999; Mercader et al., 2000), and its removal during normal development could also allow cells to rapidly respond to Shh signaling and induce gene expression. Therefore, retinoic acid could cooperate with Shh in the posterior region of the developing limb bud to relieve transcriptional repression by Gli3. This could provide a reason why Shh is required for only 12 hr during the earliest stages of wing development (Towers et al., 2011; Pickering and Towers, 2016)—around the same duration that *Shh* is expressed in the anterior of wing buds treated with high concentrations of retinoic acid.

### Retinoic Acid as a Proximalizing Factor

It is unexpected that 12 hr of *Shh* expression after HH26 is sufficient to fully duplicate the pattern of digits because polarizing region grafts are unable to duplicate the digits after HH24 (Summerbell, 1974). However, this could be due to retinoic acid acting as a proximalizing factor that prevents the activation of an intrinsic distal patterning program (Mercader et al., 1999; Mercader et al., 2000; Rosello‐Diez et al., 2014; Saiz‐Lopez et al., 2015). Indeed, our microarray data revealed that *Meis2*, which encodes a transcription factor implicated in proximal patterning (Mercader et al., 1999; Mercader et al., 2000), is expressed at twice the normal levels in the anterior of retinoic‐treated wing buds compared to untreated wing buds (Supplementary Table 1). Indeed, the later that grafts of the polarizing regions were made to the anterior margins of recipient chick wing buds, the more distal in character were the duplicated elements that formed (Summerbell, 1974). Thus, when grafts were made at HH19, all elements distal to elbow were duplicated; at HH21, the digits only; at HH22/23, only the distal parts of the digits; and as mentioned, after HH24, no elements were duplicated. This follows the normal sequence in which the structures of the limb are laid down along the proximodistal axis (Saunders, 1948). As mentioned, retinoic acid is depleted from the chick wing bud by around HH20/21 (Mercader et al., 1999; Mercader et al., 2000), and the subsequent activation of the intrinsic distal program (Rosello‐Diez et al., 2014; Saiz‐Lopez et al., 2015) can explain why later grafts can duplicate only progressively more distal structures. Taken together, these considerations support the idea that retinoic acid acts as a proximalizing factor and prevents distal development. Indeed, consistent with the notion that retinoic acid modulates the transition between proximal and distal patterning, the time of its removal can alter the duration that *Shh* is intrinsically expressed in the polarizing region (Chinnaiya et al., 2014), as well as alter the time that distal *Hoxa13* expression is initiated in digit‐forming cells (Mercader et al., 2000; Rosello‐Diez et al., 2014). The switch in proximal to distal patterning is likely to be influenced by Shh itself, which activates the expression of the retinoic acid–degrading enzyme *Cyp26B1* (Probst et al., 2011).

## Experimental Procedures

### Chick Husbandry

Fertilized Bevan Brown chicken eggs were incubated and staged according to Hamilton Hamburger (Hamburger and Hamilton, 1951).

### Skeletal Staining

Embryos were fixed in 90% ethanol for 2 days and then transferred to 0.1% Alcian blue in 80% ethanol/20% acetic acid for 1 day before being cleared in 1% KOH.

### Bead Implantations

Sieved formate‐derivatized AG1‐X2 beads (150 or 200 μm in diameter, Sigma) were soaked in all‐*trans*‐retinoic acid (Sigma, 1 μg/μl^‐1^ or 5 μg/μl^‐1^ dissolved in DMSO, also Sigma) for 1 hr and then washed twice in DMEM before being grafted to the anterior margin of chick wing buds using a sharp tungsten needle. Affi‐Gel beads (Bio‐Rad) were soaked in recombinant Shh protein (10 μg/μl^‐1^, a kind gift of Prof Joy Richman) for 2 hr and implanted the same way as retinoic acid beads.

### Whole‐mount In Situ Hybridization

Embryos were fixed in 4% PFA overnight at 4 °C, dehydrated in methanol overnight at ‐20 °C, rehydrated through a methanol/PBS series, washed in PBS, then treated with proteinase K for 20 min (10 μg/ml^‐1^), washed in PBS, fixed for 30 mins in 4% PFA at room temperature, and then pre‐hybridized at 65 °C for 2 hr (50% formamide/50% 2x SSC); 1 μg of antisense DIG‐labeled (Roche) mRNA probes were added in 1 ml of hybridization buffer (50% formamide/50% 2x SSC) at 65 °C overnight. Embryos were washed twice in hybridization buffer, twice in 50:50 hybridization buffer and MAB buffer, and then twice in MAB buffer before being transferred to blocking buffer (2% blocking reagent 20% lamb serum in MAB buffer) for 2 hr at room temperature. Embryos were transferred to blocking buffer containing anti‐digoxigenin antibody (Roche 1:2000) at 4 °C overnight, then washed in MAB buffer overnight before being transferred to NTM buffer containing NBT/BCIP and mRNA distribution and visualized using a LeicaMZ16F microscope.

### Reverse‐transcription Quantitative PCR

Anterior and posterior thirds of untreated or retinoic acid–treated wing bud tissue were dissected from eight embryos. Total RNA was extracted using TRIzol Reagent (Life Technologies), purified using a PureLink RNA mini kit (Ambion), and cDNA‐prepared using SuperScript II Reverse Transcriptase (Invitrogen). qPCR was performed on an Applied Biosystems StepOne RT‐PCR machine using TaqMan Fast Advanced Master Mix (Thermo Fisher Scientific) and a TaqMan probe and primer set designed against chicken *Shh* (Gg03338766_m1, Thermo Fisher Scientific); 5 ng cDNA was used per reaction (20‐μl volume) with cycle conditions of 95 °C for 20 sec, followed by 32 cycles of 95 °C for 1 sec and 60 °C for 20 sec. All reactions were carried out in triplicate and normalized against Eukaryotic *18S rRNA* Endogenous Control expression (Thermo Fisher Scientific). Standard error mean bars were generated from the triplicate C_T_ values. Unpaired *t*‐tests measured significance of expression change between appropriate samples. Applied Biosystems StepOne Software V2.3 was used to analyze the data and generate gene expression comparisons.

### Microarray Analyses and Clustering

Retinoic‐soaked beads were implanted to the anterior margins of HH20 chick wing buds, and after 24 hr the anterior third of the wing buds was dissected using tungsten needles. Tissue was stored in RNAlater at ‐20 °C and then RNA extracted using TRIzol (Thermo Fisher Scientific). RNA was used to probe a GeneChip Chicken Genome Array comprising 38,535 features (five replicates each containing tissue from 12 experimental wing buds). Primary data has been deposited in array express (E‐MTAB‐5283). Primary data including wild‐type posterior (five replicates), wild‐type anterior (five replicates), and *talpid* ^*3*^ anterior (five replicates) were obtained from Array Express (E‐MTAB‐309; Bangs et al., 2010). The arrays used in these previous experiments were also identical Affymetrix GeneChip Chicken Genome Arrays comprising 38,535 features. A total of 20 arrays were QC‐analyzed using the arrayQualityMetrics Bioconductor package, and all samples passed using three metrics (MA plot and boxplot or heatmap) (Kauffmann and Huber, 2010). No arrays were identified as substandard or outliers. After quality control, control probes and probes not detected in any of the 20 arrays were removed from subsequent analyses. The original arrays comprised 38,535 features and after processing and filtering 25,626 features (66.5%) remained. Normalization of the 25,626 features across all arrays was achieved using the robust multi‐array average (RMA) expression measure. In pairwise comparisons (retinoic anterior vs. wild‐type anterior, wild‐type anterior vs. wild‐type posterior, and *talpid* ^*3*^ anterior vs. wild‐type anterior), the statistical cutoff was set at an adjusted *P* value of < 0.01 with a two‐fold change in gene expression. Based on the internal stability and biological metrics provided from the ClValid R package, hierarchical clustering showed favorable properties for the Dunn index and was chosen instead of K‐means, PAM, SOM, and SOTA methods; 29 gene clusters were obtained. The statistical cutoff for a gene from each of the three pairwise comparisons to be included in the clustering was set at an adjusted *P* value of < 0.0001 with a two‐fold change in gene expression.

## Supporting information

Additional supporting information may be found in the online version of this article

Supporting Information

Supporting Information

Supporting Information

Supporting Information Tables.
