# Supplementary material for: Transcriptional changes in chick wing bud polarization induced by retinoic acid
Source: Dev Dyn. 2017 Jul 24;246(9):682–90. doi: 10.1002/dvdy.24543 (PMC5601294; doi:10.1002/dvdy.24543)
Supplement: Supplementary file 4 — Supporting Information Tables. [file DVDY-246-682-s004.docx]

**Transcriptional changes in chick wing bud polarization induced by retinoic acid**

**Supplementary Data**

**TABLE 1.** Time of *Shh* Induction in Anterior Mesenchyme Following Implantation of Retinoic Acid Beads (5 μg/μl) at Stage HH20

| *Shh* | | 24 hr | 36 hr | 40 hr | 44 hr | 48 hr | 54 hr | 60 hr |
| --- | --- | --- | --- | --- | --- | --- | --- | --- |
| Undetectable | *n* | 12 | 10 | 1 | 1 | 2 | 3 | 5 |
| Weak | *n* | 0 | 1 | 0 | 1 | 3 | 3 | 1 |
| Strong | *n* | 0 | 1 | 6 | 7 | 3 | 1 | 0 |

**TABLE 2.** Digit Patterns Following Retinoic Acid (5 μg/μl) and Cyclopamine Treatment at Stage HH20

| Retinoic acid (1μg/μl) | *n* | Retinoic acid  (5μg/μl) | *n* | Cyclopamine | *n* | Retinoic acid  (5 μg/μl) +cyclopamine | *n* |
| --- | --- | --- | --- | --- | --- | --- | --- |
| 32123 | 3 | 323 | 8 | 12 | 5 | 12 | 4 |
| 21123 | 3 | 3223 | 7 | 12/3 | 3 | 12/3 | 1 |
| 2123 | 2 | 32123 | 8 |  |  | n123 | 1 |
| 1123 | 3 | 2123 | 2 |  |  |  |  |
|  |  | 2123 | 1 |  |  |  |  |

2/3, fused digits; n, nubbin

**TABLE 3.** Digit Patterns Following Retinoic Acid Treatment (Beads Soaked in 5 μg/μl Retinoic Acid) at Stage HH20 (Same Replicate Pools as Used for Microarray Analysis)

| Experiment A | *n* | Experiment  B | *n* | Experiment C | *n* | Experiment D | *n* | Experiment E | *n* |
| --- | --- | --- | --- | --- | --- | --- | --- | --- | --- |
| 123 | 1 | 3223 | 2 | 3223 | 1 | 323 | 1 | 323 | 1 |
| 1123 | 1 | 21123 | 1 | 32123 | 1 | Truncated wing | 1 | 321123 | 2 |
| 3223 | 1 | 32123 | 2 |  |  | 123 | 1 | 1123 | 1 |
| 32123 | 2 |  |  |  |  | 2123 | 2 |  |  |
|  |  |  |  |  |  | 3223 | 1 |  |  |
