# Supplementary material for: Transcriptional changes in chick wing bud polarization induced by retinoic acid
Source: Dev Dyn. 2017 Jul 24;246(9):682–90. doi: 10.1002/dvdy.24543 (PMC5601294; doi:10.1002/dvdy.24543)
Supplement: Supplementary file 4 [file DVDY-246-682-s003.docx]

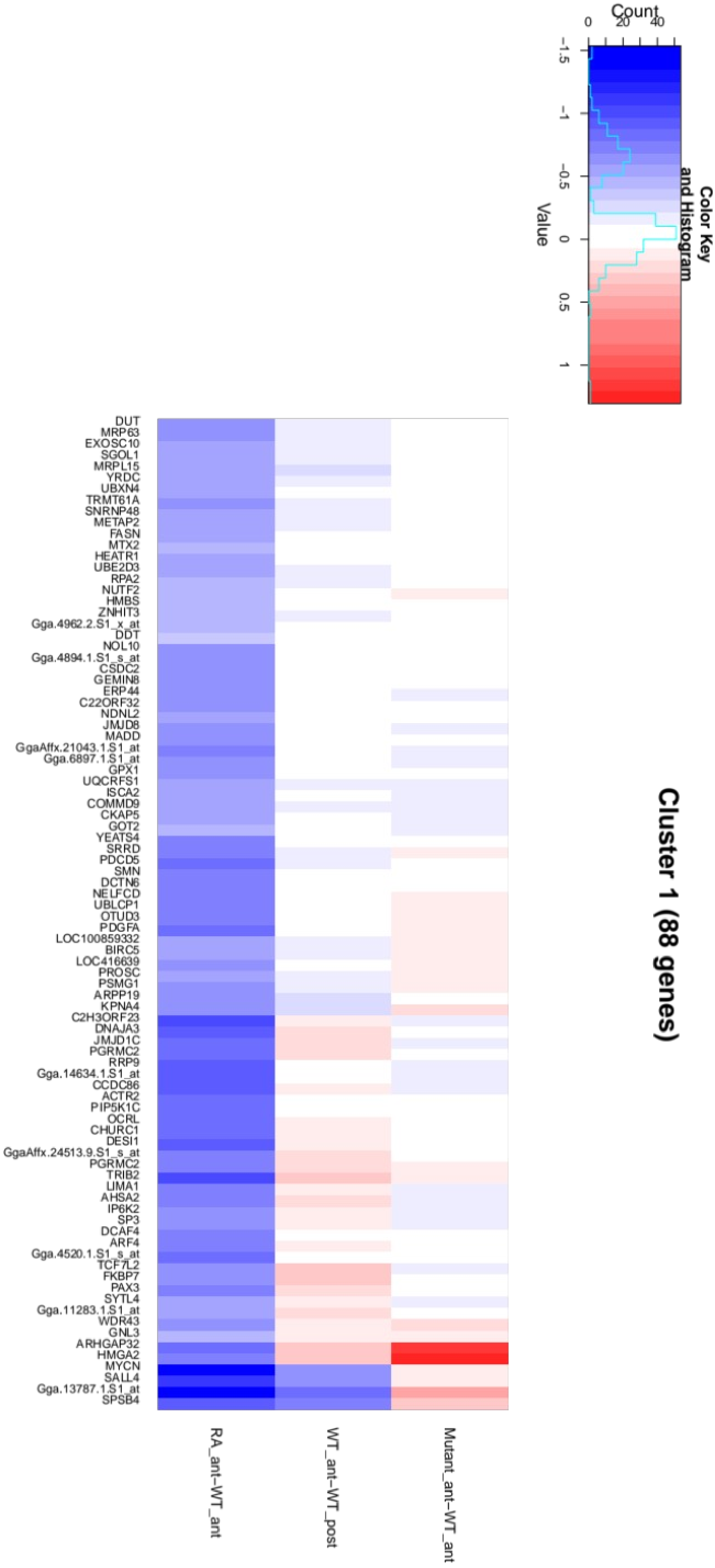

Cluster 1 (88 genes)
Color Key
and Histogram
Count
0
20
40
-1.5
-1
-0.5
0
0.5
1
Value
DUT
MRP63
EXOSC10
SGOL1
MRPL15
YRDC
UBXN4
TRMT61A
SNRNP48
METAP2
FASN
MTX2
HEATR1
UBE2D3
RPA2
NUTF2
HMBS
ZNHIT3
Gga.4962.2.S1_x_at
DDT
NOL10
Gga.4894.1.S1_s_at
CSDC2
GEMIN8
ERP44
C22ORF32
NDNL2
JMJD8
MADD
GgaAffx.21043.1.S1_at
Gga.6897.1.S1_at
GPX1
UQCRFS1
ISCA2
COMMD9
CKAP5
GOT2
YEATS4
SRRD
PDCD5
SMN
DCTN6
NELFCD
UBLCP1
OTUD3
PDGFA
LOC100859332
BIRC5
LOC416639
PROSC
PSMG1
ARPP19
KPNA4
C2H3ORF23
DNAJA3
JMJD1C
PGRMC2
RRP9
Gga.14634.1.S1_at
CCDC86
ACTR2
PIP5K1C
OCRL
CHURC1
DESI1
GgaAffx.24513.9.S1_s_at
PGRMC2
TRIB2
LIMA1
AHSA2
IP6K2
SP3
DCAF4
ARF4
Gga.4520.1.S1_s_at
TCF7L2
FKBP7
PAX3
SYTL4
Gga.11283.1.S1_at
WDR43
GNL3
ARHGAP32
HMGA2
MYCN
SALL4
Gga.13787.1.S1_at
SPSB4
RA_ant-WT_ant
WT_ant-WT_post
Mutant_ant-WT_ant

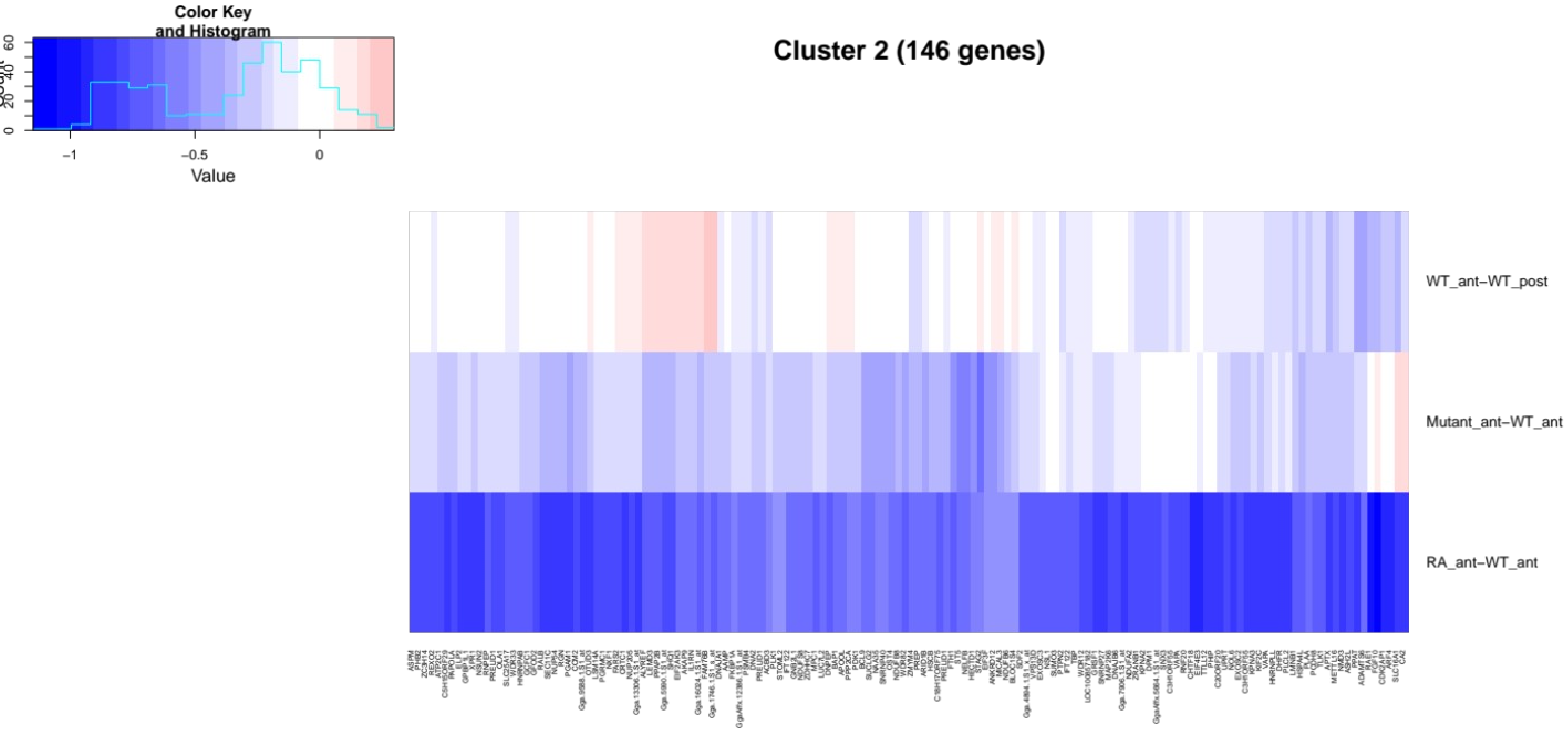
Color Key
and Histogram
Count
0
20
40
60
−1
−0.5
0
Value
Cluster 2 (146 genes)
WT_ant−WT_post
Mutant_ant−WT_ant
RA_ant−WT_ant

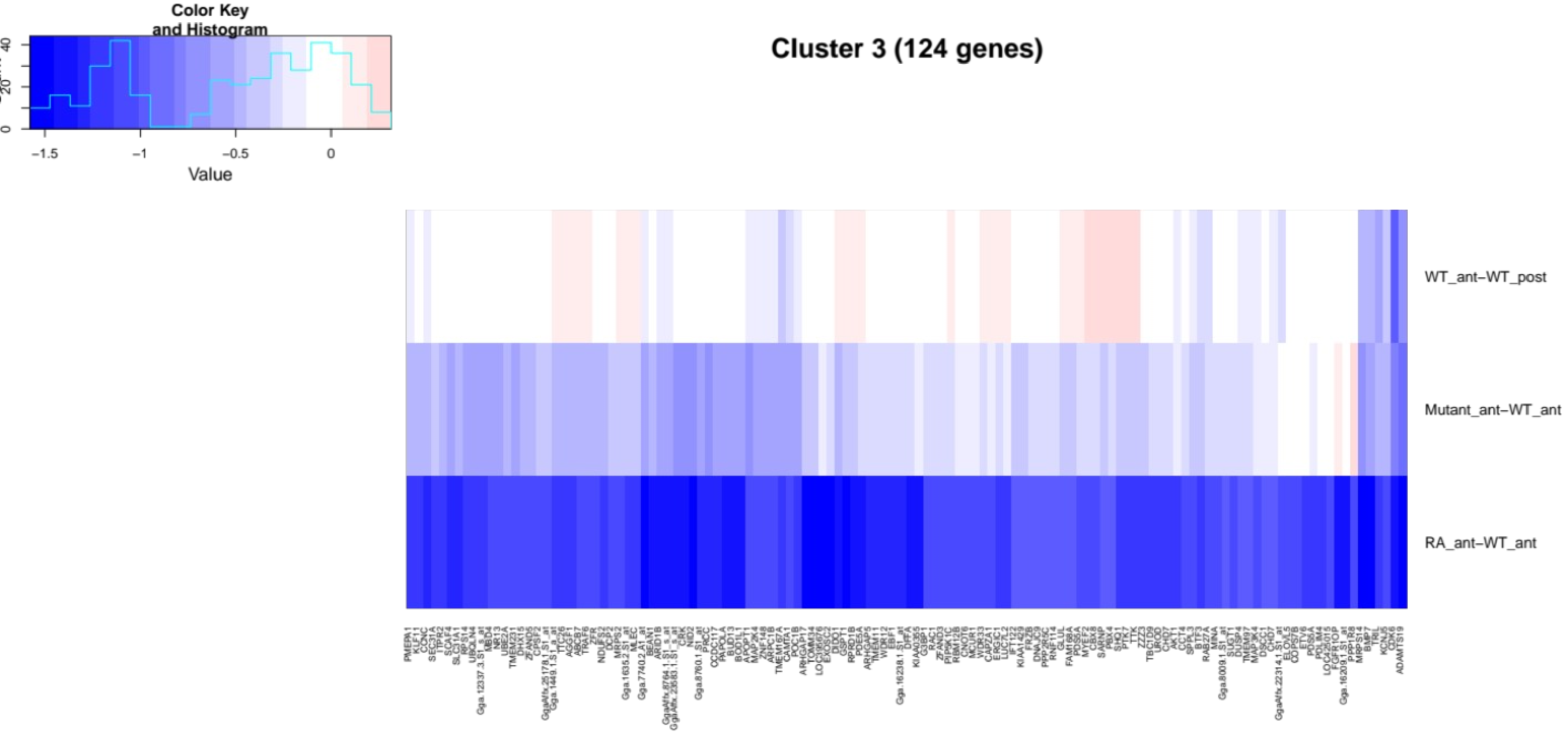

Cluster 3 (124 genes)
Color Key and Histogram
Count
0
20
40
−1.5
−1
−0.5
0
Value
WT_ant−WT_post
Mutant_ant−WT_ant
RA_ant−WT_ant
PMEPA1
KLF11
CCNC
SEC31A
ITPR2
SCAF4
SLC31A1
RPS14
UBQLN4
Gga.12337.3.S1_s_at
MBD4
NR13
UBE2A
TMEM231
DHX15
ZFAND5
CPSF2
GgaAffx.25178.1.S1_at
Gga.1449.1.S1_a_at
TTC26
AGGF1
ABCB7
TRAF6
ZFR
NDUFS2
DCP2
MRPS2
Gga.1635.2.S1_at
MLEC
Gga.7740.2.A1_at
BEAN1
ARID1B
GgaAffx.8764.1.S1_s_at
GgaAffx.23583.1.S1_s_at
CRK
NID2
Gga.8760.1.S1_at
PRCC
CCDC117
PAPOLA
BUD13
BOD1L1
APOPT1
MAP2K4
ZNF148
ARPC1B
TMEM167A
CAMTA1
POC1B
ARHGAP17
TOMM34
LOC395676
EXOSC2
DIDO1
GSPT1
RPRD1B
PDE5A
ARHGAP5
TMEM11
WDR12
EBF1
Gga.16238.1.S1_at
DFFA
KIAA0355
G3BP1
RAC1
ZFAND3
PIP5K1C
RBM12B
CNOT6
MCUR1
WDR33
CAPZA1
ERGIC1
LUC7L2
IFT122
KIAA1429
FRZB
DNAJC9
PPP2R5C
RNF114
GLUL
FAM168A
PDS5A
MYEF2
CBX8
SARNP
PBX4
SHQ1
PTK7
TTK
ZZZ3
TBC1D9
UROD
CHD7
AKT1
CCT4
SPPL3
BTF3
RAB27A
MINA
Gga.8009.1.S1_at
SUGT1
DUSP4
TMEM97
MAP3K4
DSCC1
CHD7
GgaAffx.22314.1.S1_at
ELOVL5
COPS7B
ETV6
PDS5A
PDLIM4
LOC425015
FGFR1OP
Gga.16209.1.S1_at
PPP1R8
MRPS14
BMP7
TRIL
KCNJ5
CDK6
ADAMTS19

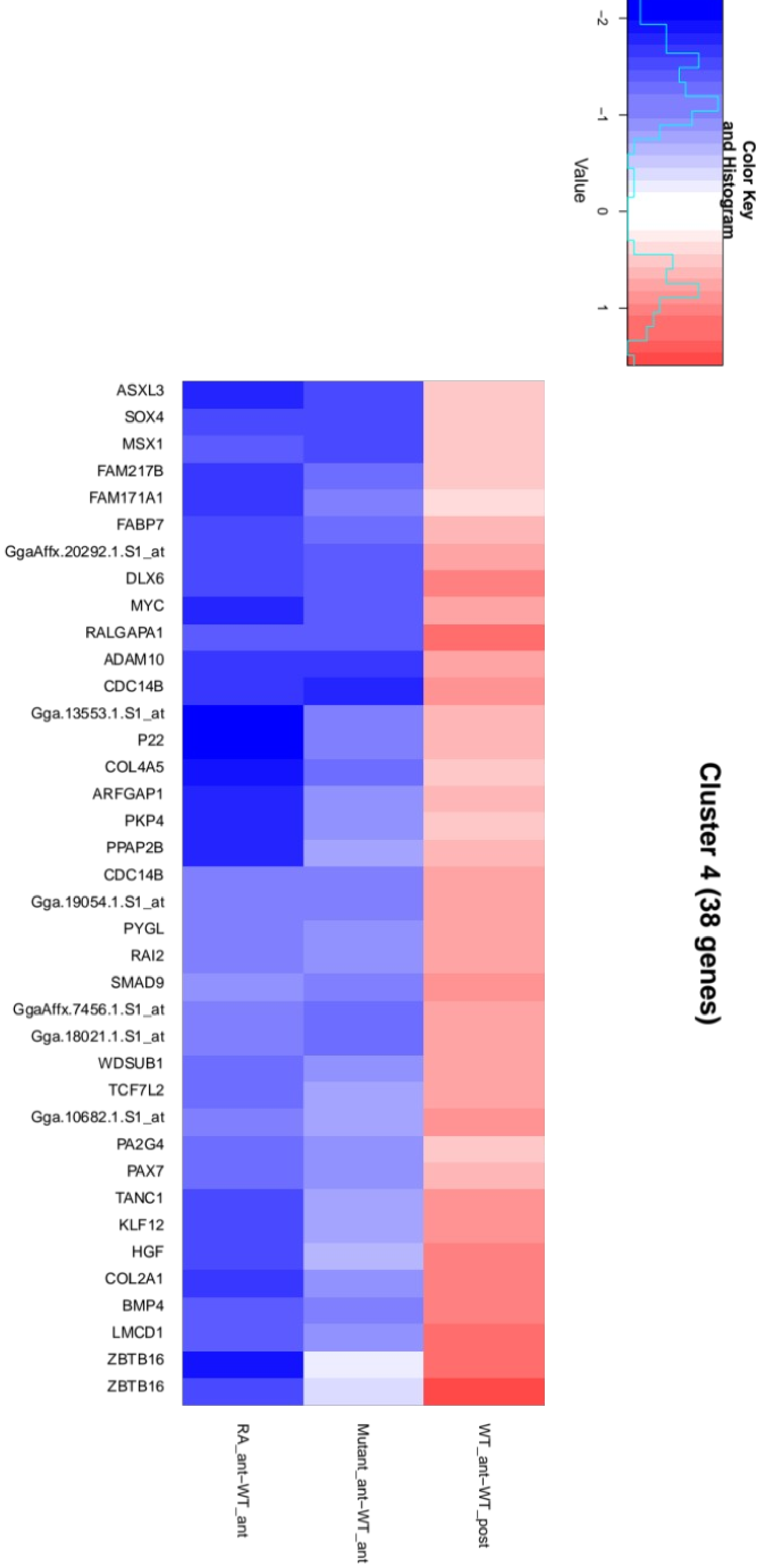
Color Key
and Histogram
Value
-2
-1
0
1
Cluster 4 (38 genes)
ASXL3
SOX4
MSX1
FAM217B
FAM171A1
FABP7
GgaAffx.20292.1.S1_at
DLX6
MYC
RALGAPA1
ADAM10
CDC14B
Gga.13553.1.S1_at
P22
COL4A5
ARFGAP1
PKP4
PPAP2B
CDC14B
Gga.19054.1.S1_at
PYGL
RAI2
SMAD9
GgaAffx.7456.1.S1_at
Gga.18021.1.S1_at
WDSUB1
TCF7L2
Gga.10682.1.S1_at
PA2G4
PAX7
TANC1
KLF12
HGF
COL2A1
BMP4
LMCD1
ZBTB16
ZBTB16
RA_ant-WT_ant
Mutant_ant-WT_ant
WT_ant-WT_post

Cluster 5 (77 genes)

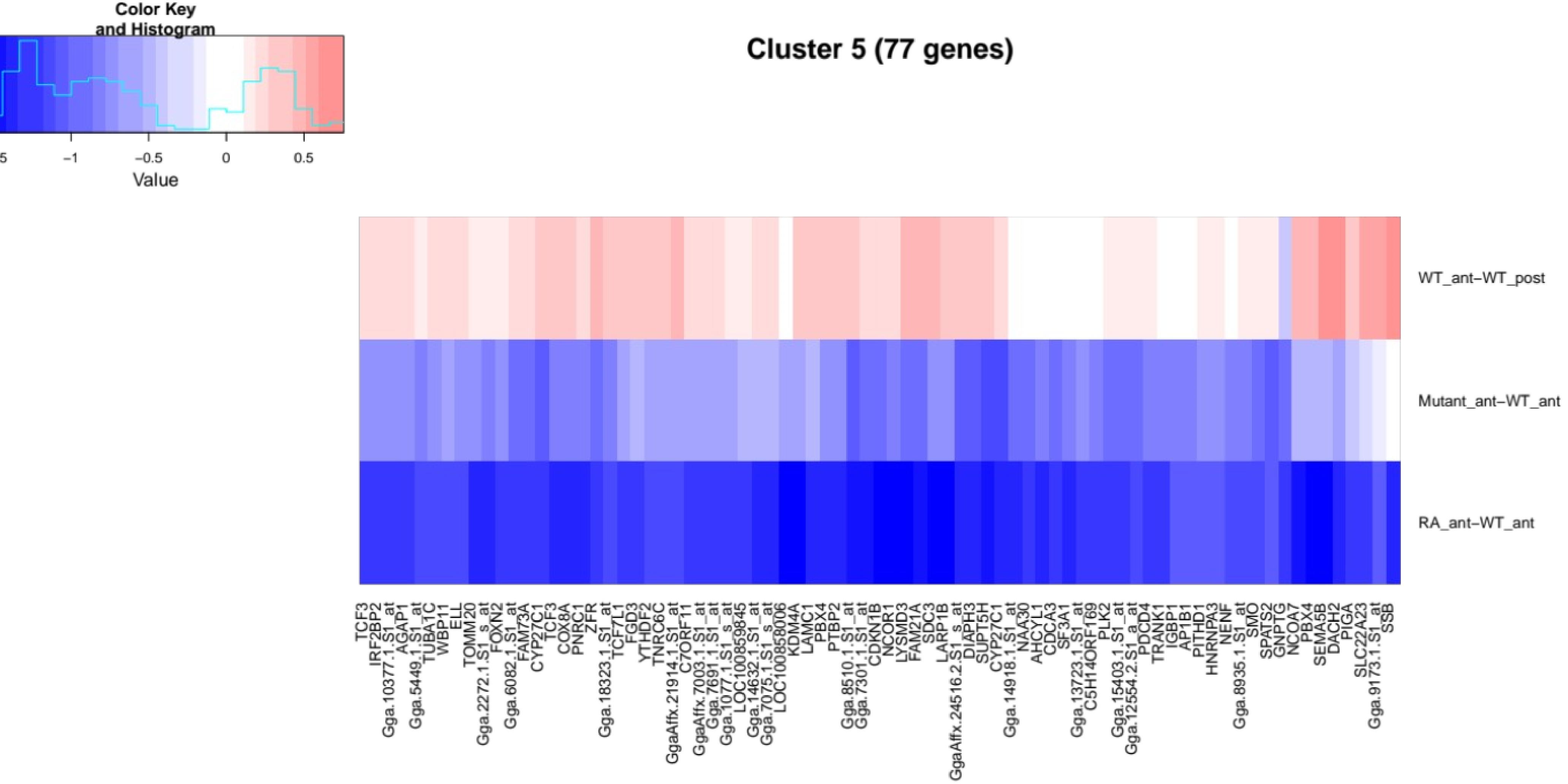

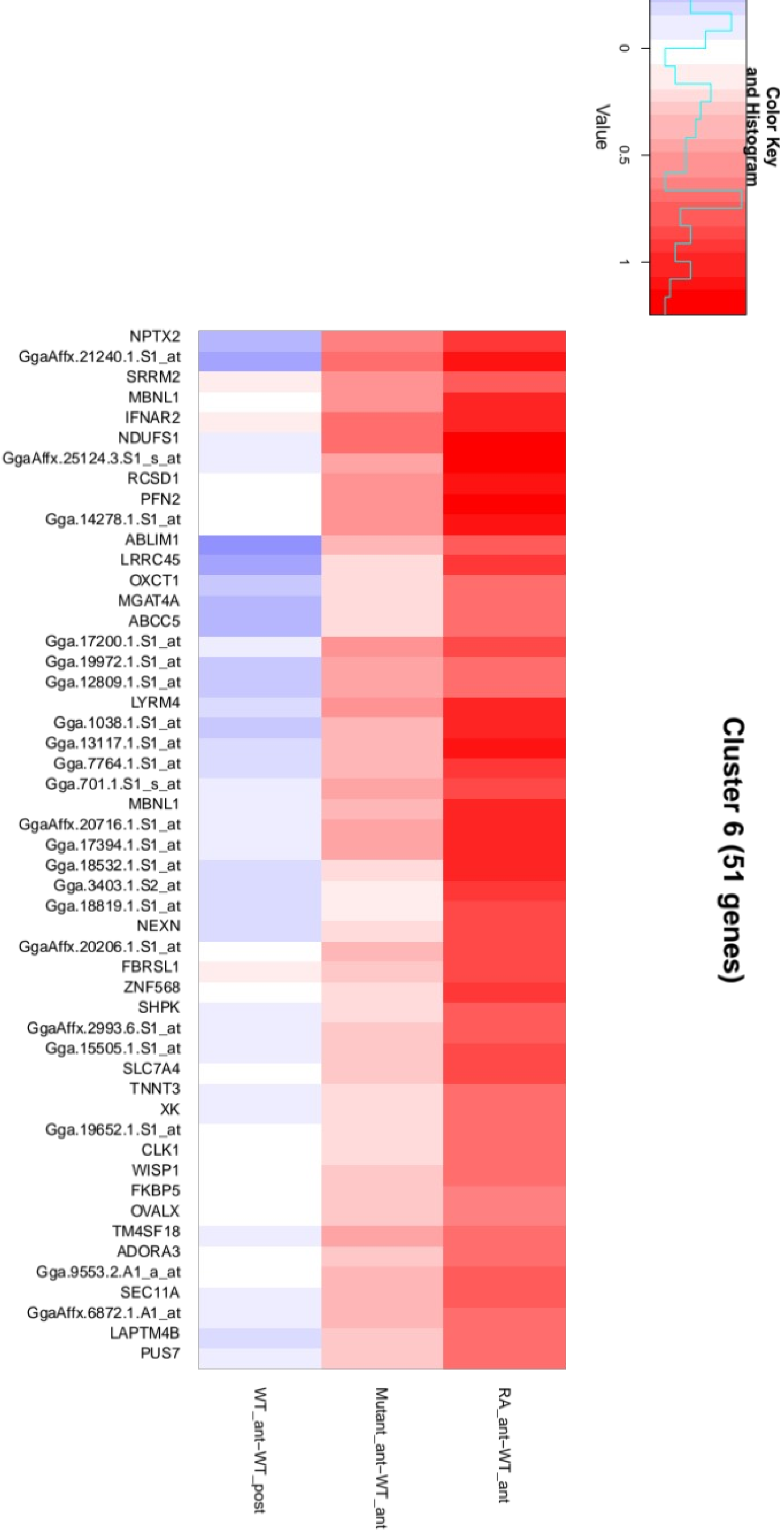

Color Key
and Histogram
Value
0
0.5
1
Cluster 6 (51 genes)
NPTX2
GgaAffx.21240.1.S1_at
SRRM2
MBNL1
IFNAR2
NDUFS1
GgaAffx.25124.3.S1_s_at
RCSD1
PFN2
Gga.14278.1.S1_at
ABLIM1
LRRC45
OXCT1
MGAT4A
ABCC5
Gga.17200.1.S1_at
Gga.19972.1.S1_at
Gga.12809.1.S1_at
LYRM4
Gga.1038.1.S1_at
Gga.13117.1.S1_at
Gga.7764.1.S1_at
Gga.701.1.S1_s_at
MBNL1
GgaAffx.20716.1.S1_at
Gga.17394.1.S1_at
Gga.18532.1.S1_at
Gga.3403.1.S2_at
Gga.18819.1.S1_at
NEXN
GgaAffx.20206.1.S1_at
FBRSL1
ZNF568
SHPK
GgaAffx.2993.6.S1_at
Gga.15505.1.S1_at
SLC7A4
TNNT3
XK
Gga.19652.1.S1_at
CLK1
WISP1
FKBP5
OVALX
TM4SF18
ADORA3
Gga.9553.2.A1_a_at
SEC11A
GgaAffx.6872.1.A1_at
LAPTM4B
PUS7
WT_ant−WT_post
Mutant_ant−WT_ant
RA_ant−WT_ant

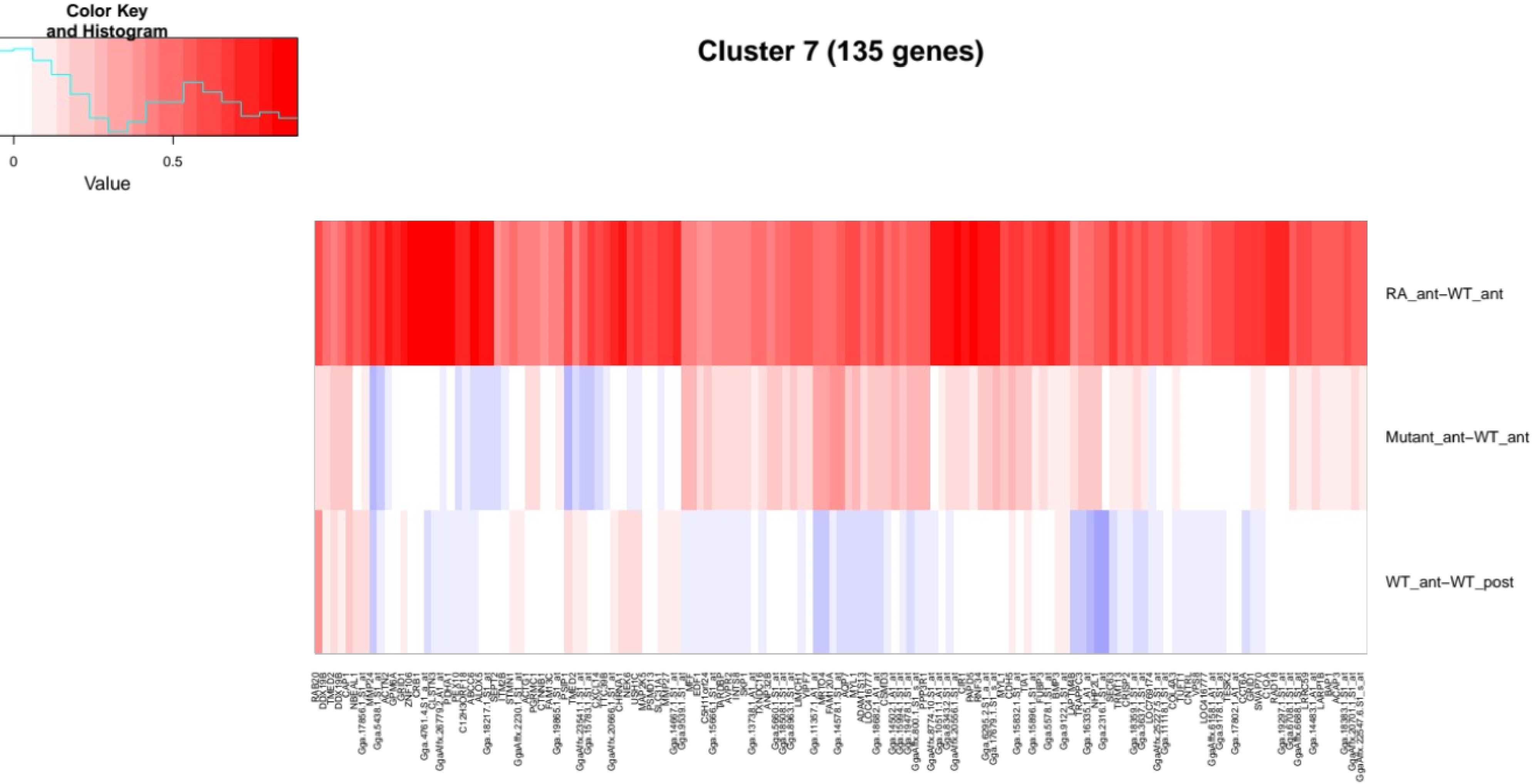
Cluster 7 (135 genes)
Color Key
and Histogram
0
0.5
Value
RA_ant−WT_ant
Mutant_ant−WT_ant
WT_ant−WT_post

Cluster 8 (84 genes)

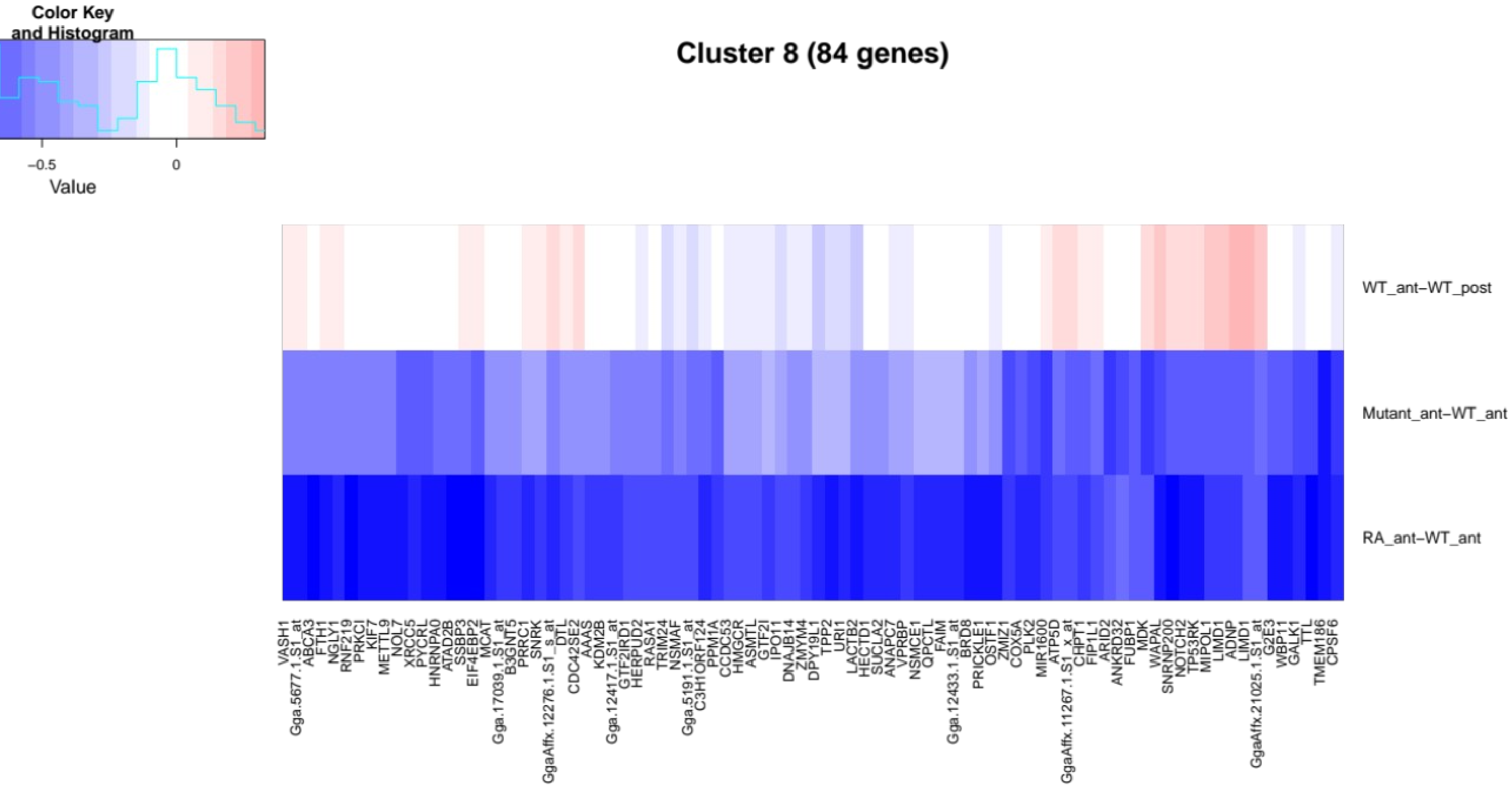

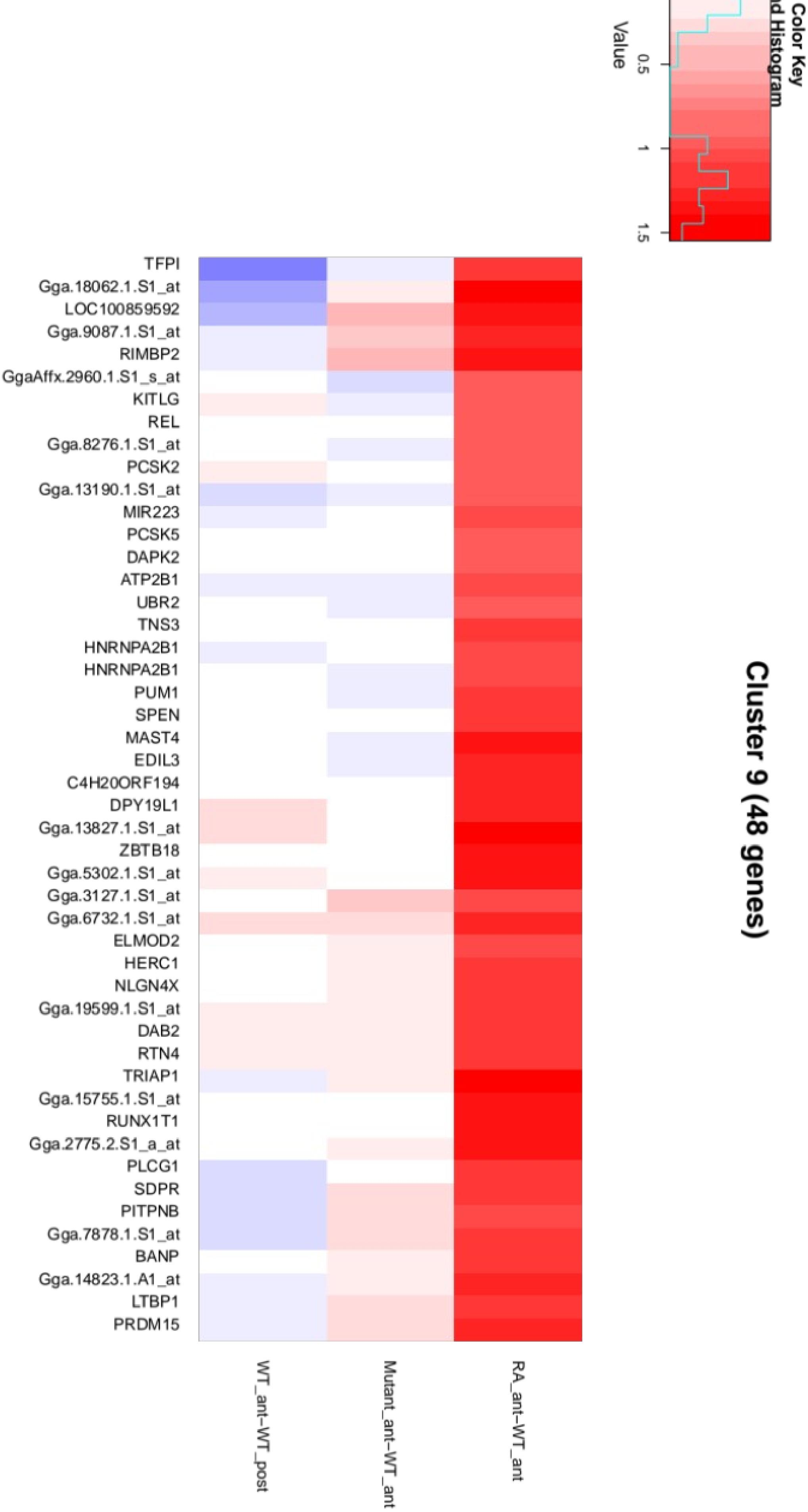

Cluster 9 (48 genes)
Color Key and Histogram
Value
0.5
1
1.5
TFPI
Gga.18062.1.S1_at
LOC100859592
Gga.9087.1.S1_at
RIMBP2
GgaAffx.2960.1.S1_s_at
KITLG
REL
Gga.8276.1.S1_at
PCSK2
Gga.13190.1.S1_at
MIR223
PCSK5
DAPK2
ATP2B1
UBR2
TNS3
HNRNPA2B1
HNRNPA2B1
PUM1
SPEN
MAST4
EDIL3
C4H20ORF194
DPY19L1
Gga.13827.1.S1_at
ZBTB18
Gga.5302.1.S1_at
Gga.3127.1.S1_at
Gga.6732.1.S1_at
ELMOD2
HERC1
NLGN4X
Gga.19599.1.S1_at
DAB2
RTN4
TRIAP1
Gga.15755.1.S1_at
RUNX1T1
Gga.2775.2.S1_a_at
PLCG1
SDPR
PITPNB
Gga.7878.1.S1_at
BANP
Gga.14823.1.A1_at
LTBP1
PRDM15
WT_ant−WT_post
Mutant_ant−WT_ant
RA_ant−WT_ant

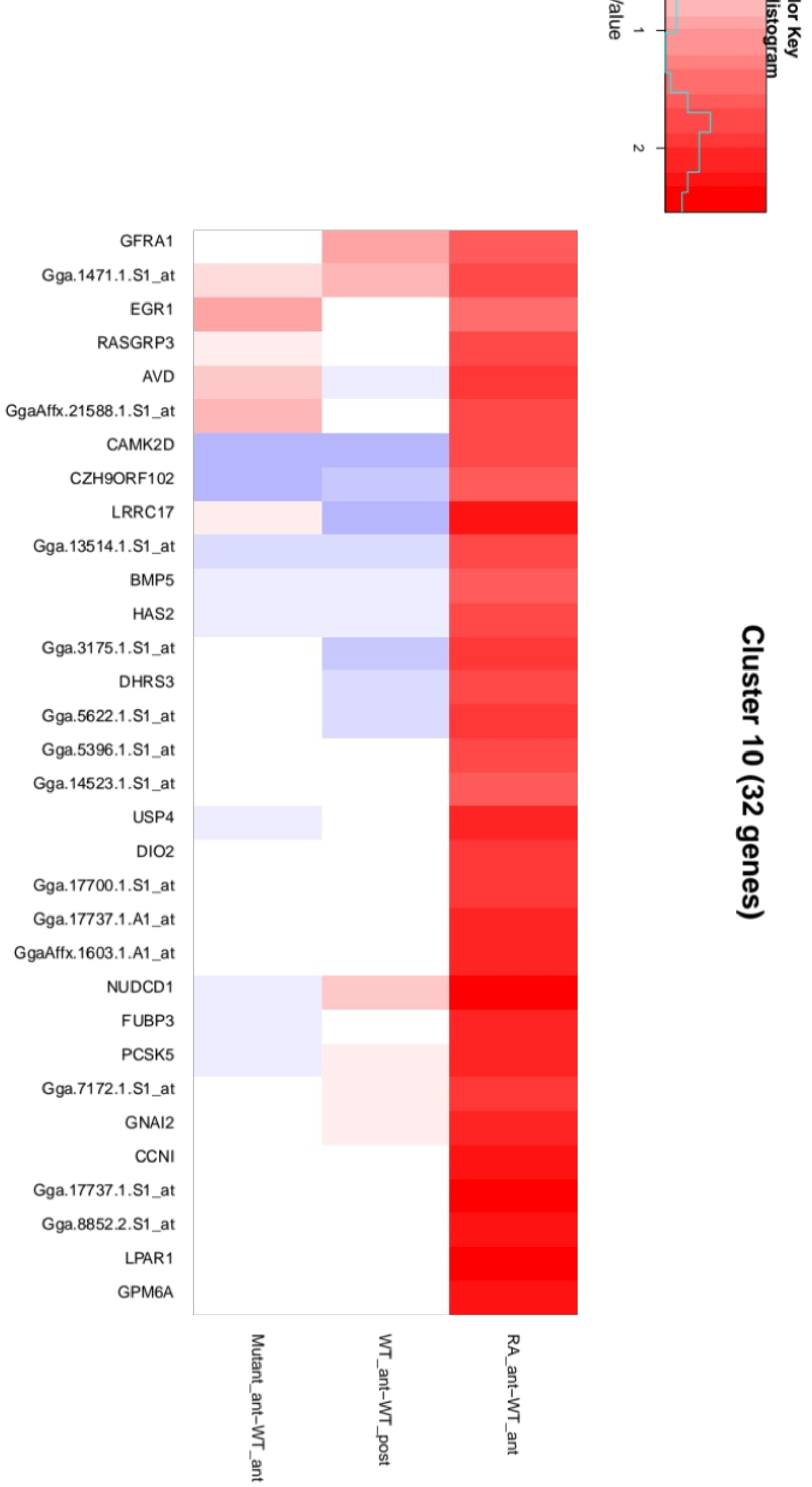
lor Key
listogram
/alue
1
2
Cluster 10 (32 genes)
GFRA1
Gga.1471.1.S1_at
EGR1
RASGRP3
AVD
GgaAffx.21588.1.S1_at
CAMK2D
CZH9ORF102
LRRC17
Gga.13514.1.S1_at
BMP5
HAS2
Gga.3175.1.S1_at
DHRS3
Gga.5622.1.S1_at
Gga.5396.1.S1_at
Gga.14523.1.S1_at
USP4
DIO2
Gga.17700.1.S1_at
Gga.17737.1.A1_at
GgaAffx.1603.1.A1_at
NUDCD1
FUBP3
PCSK5
Gga.7172.1.S1_at
GNAI2
CCNI
Gga.17737.1.S1_at
Gga.8852.2.S1_at
LPAR1
GPM6A
Mutant_ant–WT_ant
WT_ant–WT_post
RA_ant–WT_ant

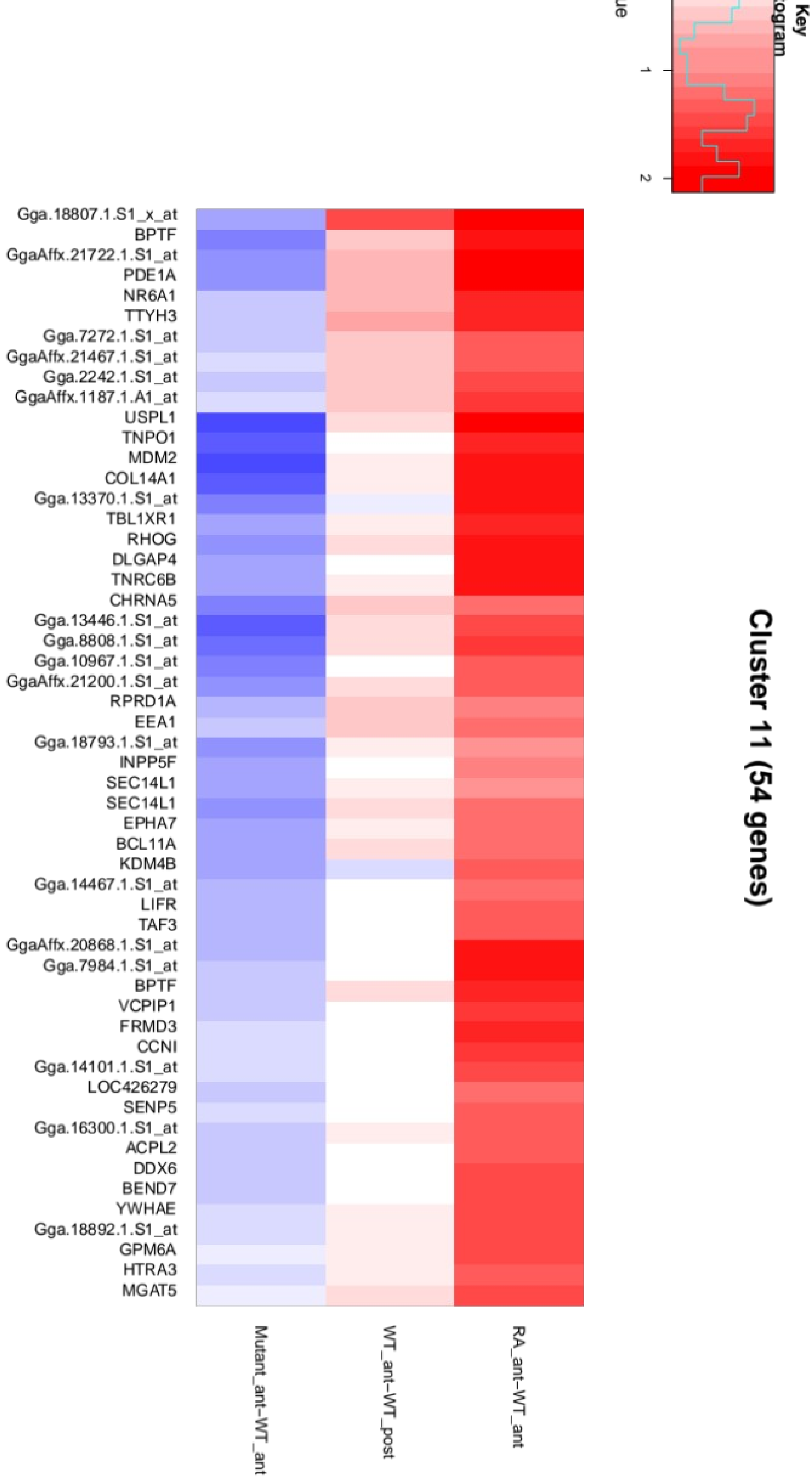
Cluster 11 (54 genes)
Key
Histogram
Value
1
2
Gga.18807.1.S1_x_at
BPTF
GgaAffx.21722.1.S1_at
PDE1A
NR6A1
TTYH3
Gga.7272.1.S1_at
GgaAffx.21467.1.S1_at
Gga.2242.1.S1_at
GgaAffx.1187.1.A1_at
USPL1
TNPO1
MDM2
COL14A1
Gga.13370.1.S1_at
TBL1XR1
RHOG
DLGAP4
TNRC6B
CHRNA5
Gga.13446.1.S1_at
Gga.8808.1.S1_at
Gga.10967.1.S1_at
GgaAffx.21200.1.S1_at
RPRD1A
EEA1
Gga.18793.1.S1_at
INPP5F
SEC14L1
SEC14L1
EPHA7
BCL11A
KDM4B
Gga.14467.1.S1_at
LIFR
TAF3
GgaAffx.20868.1.S1_at
Gga.7984.1.S1_at
BPTF
VCPIP1
FRMD3
CCNI
Gga.14101.1.S1_at
LOC426279
SENP5
Gga.16300.1.S1_at
ACPL2
DDX6
BEND7
YWHAE
Gga.18892.1.S1_at
GPM6A
HTRA3
MGAT5
Mutant_ant–WT_ant
WT_ant–WT_post
RA_ant–WT_ant

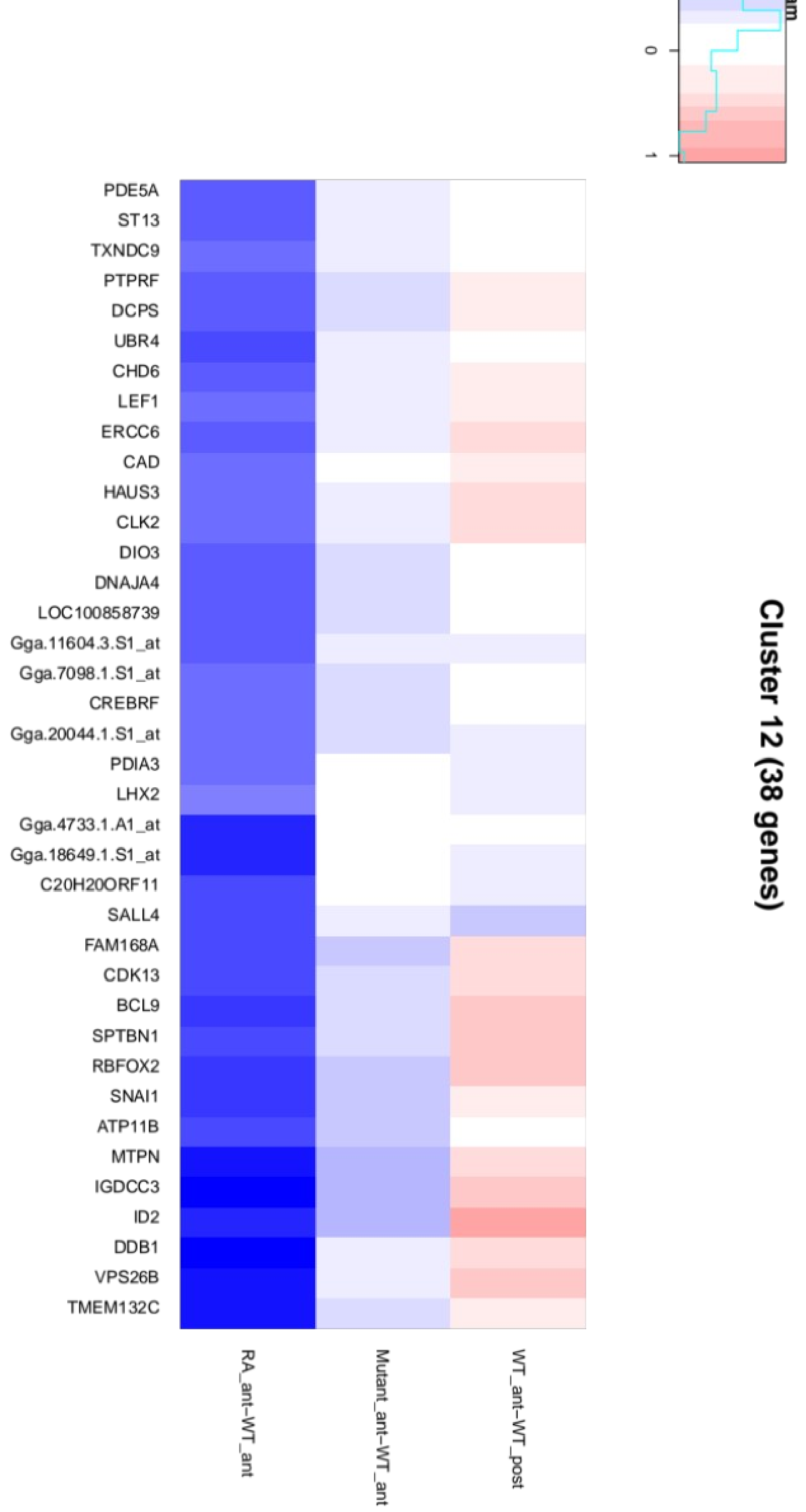
Cluster 12 (38 genes)
PDE5A
ST13
TXNDC9
PTPRF
DCPS
UBR4
CHD6
LEF1
ERCC6
CAD
HAUS3
CLK2
DIO3
DNAJA4
LOC100858739
Gga.11604.3.S1_at
Gga.7098.1.S1_at
CREBRF
Gga.20044.1.S1_at
PDIA3
LHX2
Gga.4733.1.A1_at
Gga.18649.1.S1_at
C20H20ORF11
SALL4
FAM168A
CDK13
BCL9
SPTBN1
RBFOX2
SNAI1
ATP11B
MTPN
IGDCC3
ID2
DDB1
VPS26B
TMEM132C
RA_ant−WT_ant
Mutant_ant−WT_ant
WT_ant−WT_post
0
1

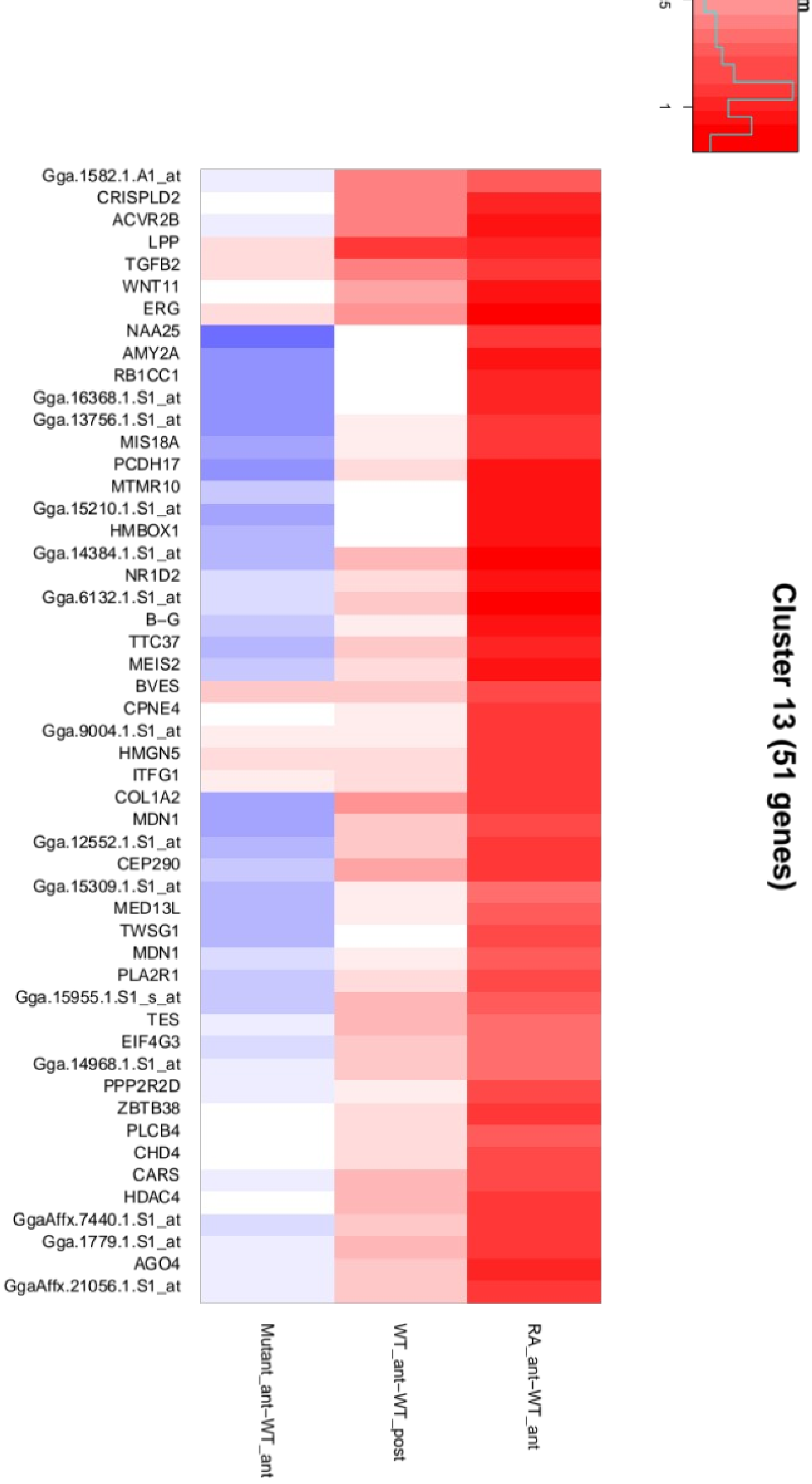
Cluster 13 (51 genes)
Gga.1582.1.A1_at
CRISPLD2
ACVR2B
LPP
TGFB2
WNT11
ERG
NAA25
AMY2A
RB1CC1
Gga.16368.1.S1_at
Gga.13756.1.S1_at
MIS18A
PCDH17
MTMR10
Gga.15210.1.S1_at
HMBOX1
Gga.14384.1.S1_at
NR1D2
Gga.6132.1.S1_at
B-G
TTC37
MEIS2
BVES
CPNE4
Gga.9004.1.S1_at
HMGN5
ITFG1
COL1A2
MDN1
Gga.12552.1.S1_at
CEP290
Gga.15309.1.S1_at
MED13L
TWSG1
MDN1
PLA2R1
Gga.15955.1.S1_s_at
TES
EIF4G3
Gga.14968.1.S1_at
PPP2R2D
ZBTB38
PLCB4
CHD4
CARS
HDAC4
GgaAffx.7440.1.S1_at
Gga.1779.1.S1_at
AGO4
GgaAffx.21056.1.S1_at
Mutant_ant-WT_ant
WT_ant-WT_post
RA_ant-WT_ant

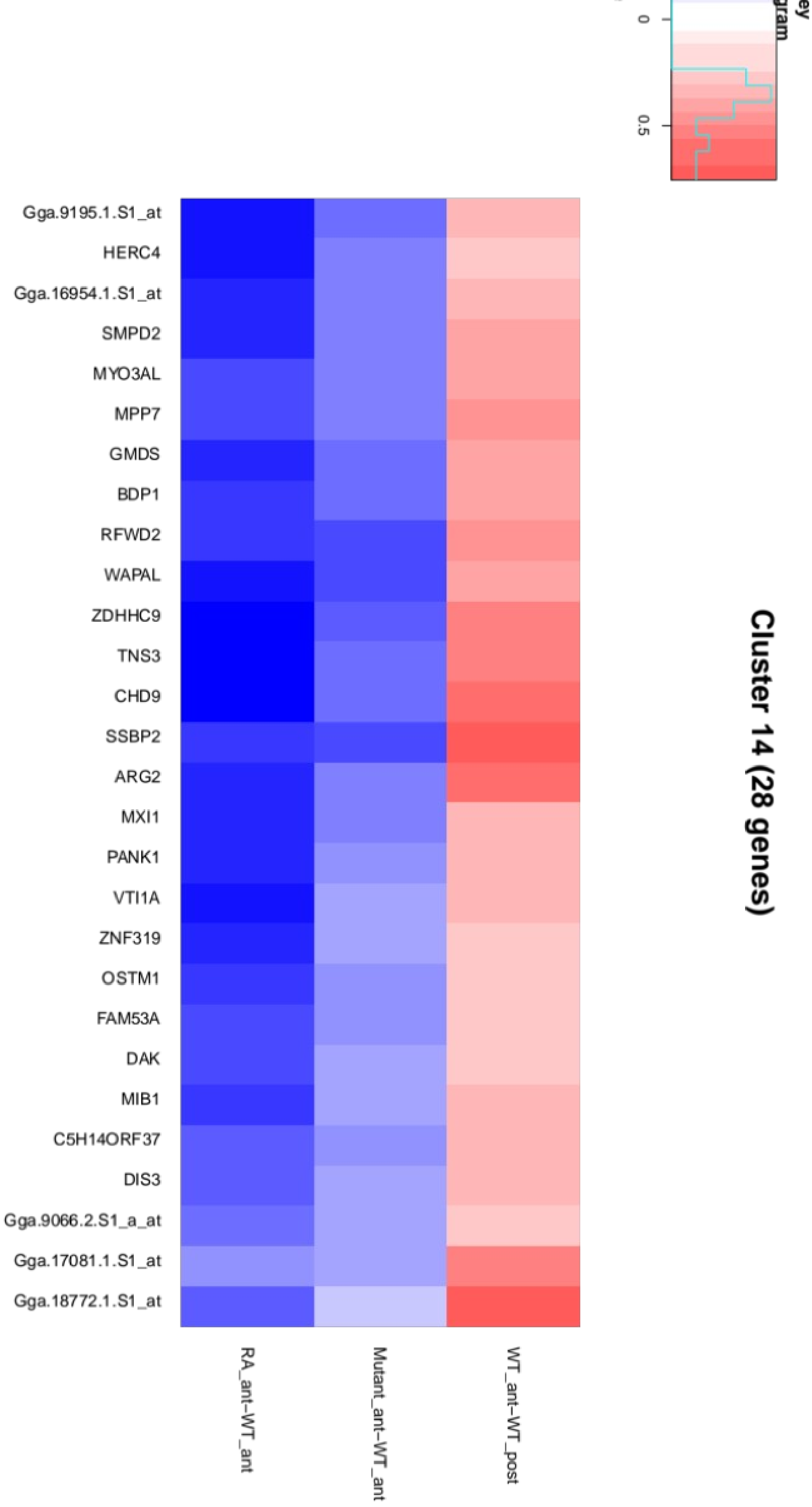
Cluster 14 (28 genes)
0
0.5
Gga.9195.1.S1_at
HERC4
Gga.16954.1.S1_at
SMPD2
MYO3AL
MPP7
GMDS
BDP1
RFWD2
WAPAL
ZDHHC9
TNS3
CHD9
SSBP2
ARG2
MXI1
PANK1
VTI1A
ZNF319
OSTM1
FAM53A
DAK
MIB1
C5H14ORF37
DIS3
Gga.9066.2.S1_a_at
Gga.17081.1.S1_at
Gga.18772.1.S1_at
RA_ant−WT_ant
Mutant_ant−WT_ant
WT_ant−WT_post

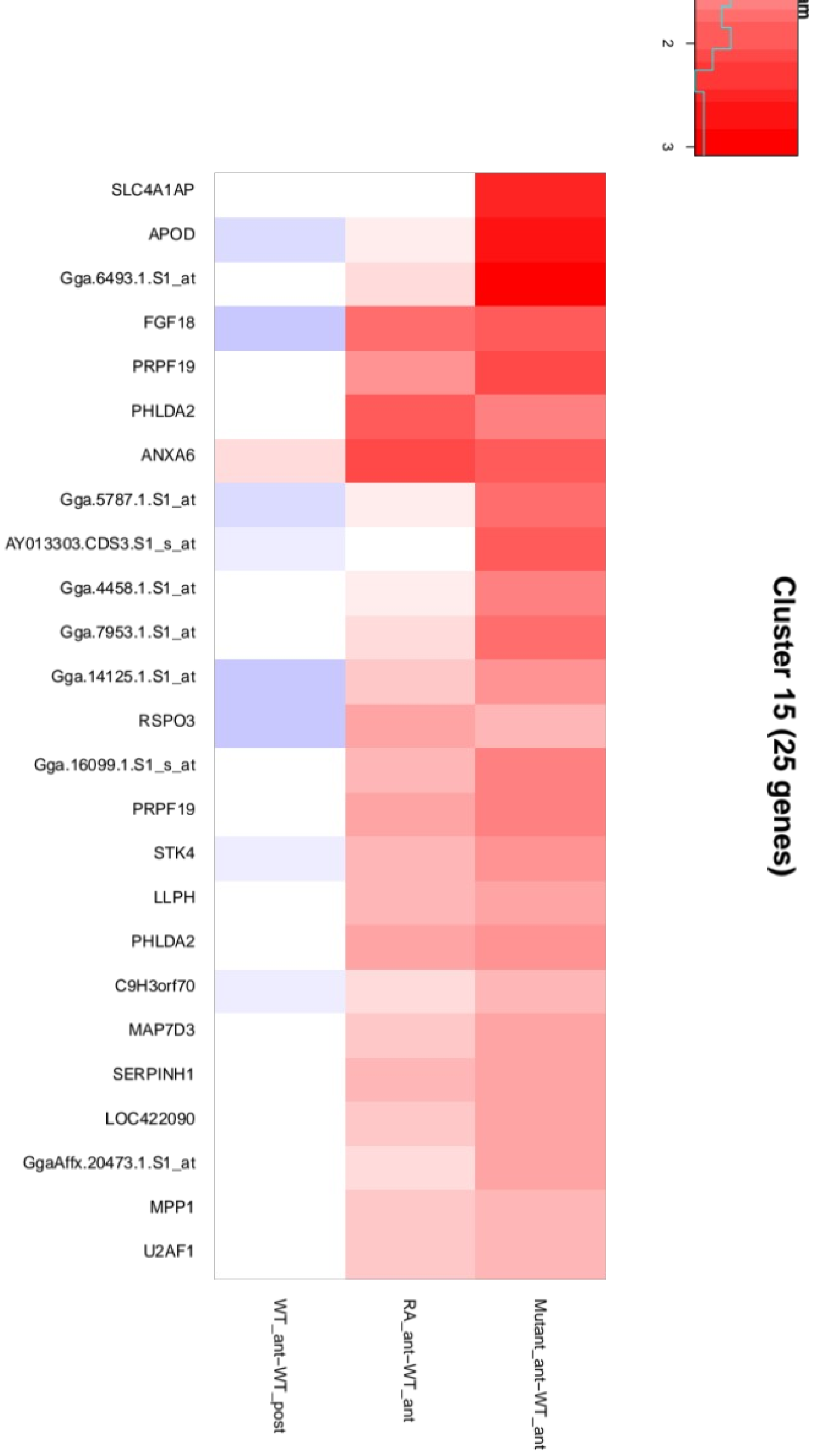

SLC4A1AP
APOD
Gga.6493.1.S1_at
FGF18
PRPF19
PHLDA2
ANXA6
Gga.5787.1.S1_at
AY013303.CDS3.S1_s_at
Gga.4458.1.S1_at
Gga.7953.1.S1_at
Gga.14125.1.S1_at
RSPO3
Gga.16099.1.S1_s_at
PRPF19
STK4
LLPH
PHLDA2
C9H3orf70
MAP7D3
SERPINH1
LOC422090
GgaAffx.20473.1.S1_at
MPP1
U2AF1
WT_ant−WT_post
RA_ant−WT_ant
Mutant_ant−WT_ant
Cluster 15 (25 genes)
2
3

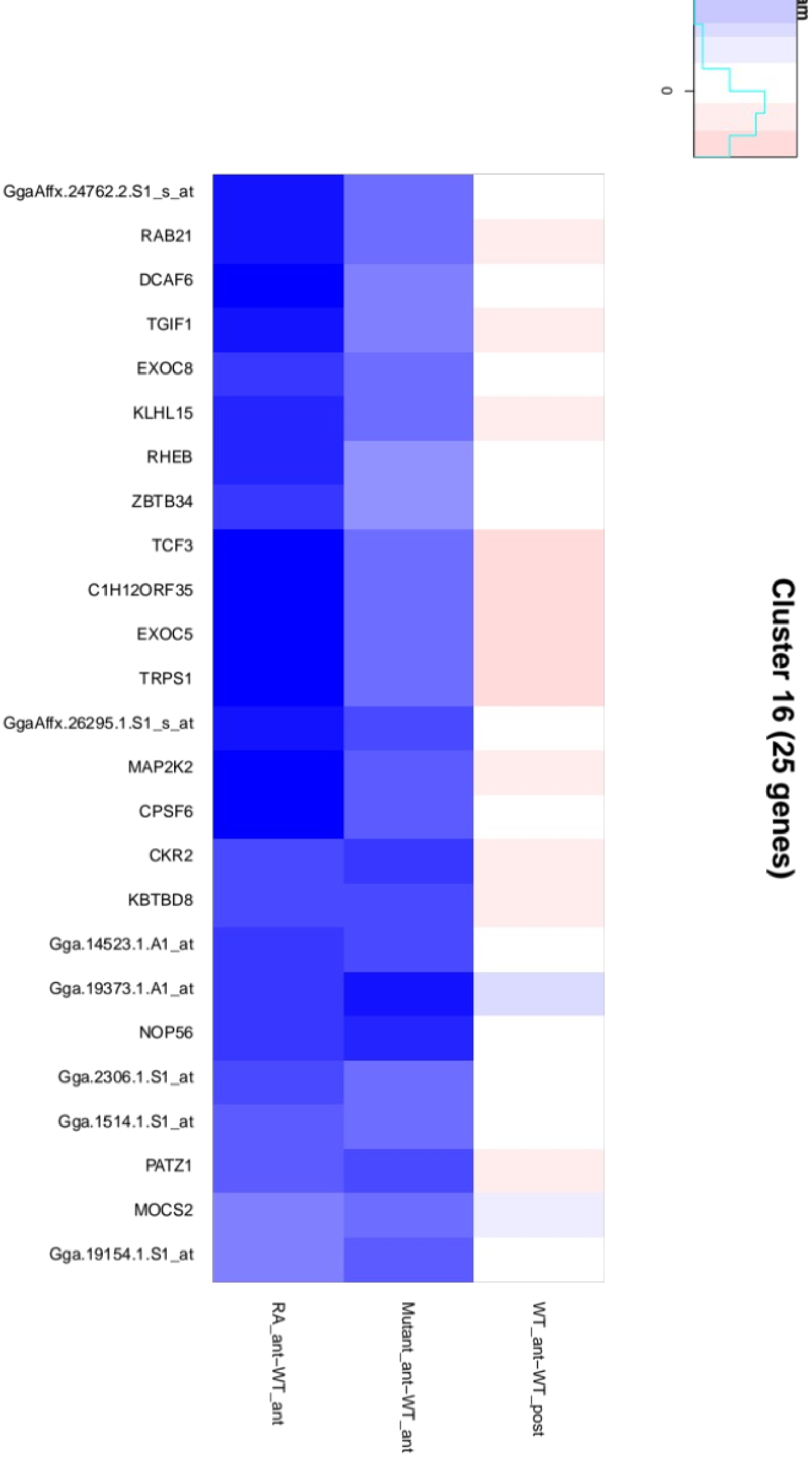
Cluster 16 (25 genes)
0
GgaAffx.24762.2.S1_s_at
RAB21
DCAF6
TGIF1
EXOC8
KLHL15
RHEB
ZBTB34
TCF3
C1H12ORF35
EXOC5
TRPS1
GgaAffx.26295.1.S1_s_at
MAP2K2
CPSF6
CKR2
KBTBD8
Gga.14523.1.A1_at
Gga.19373.1.A1_at
NOP56
Gga.2306.1.S1_at
Gga.1514.1.S1_at
PATZ1
MOCS2
Gga.19154.1.S1_at
RA_ant−WT_ant
Mutant_ant−WT_ant
WT_ant−WT_post

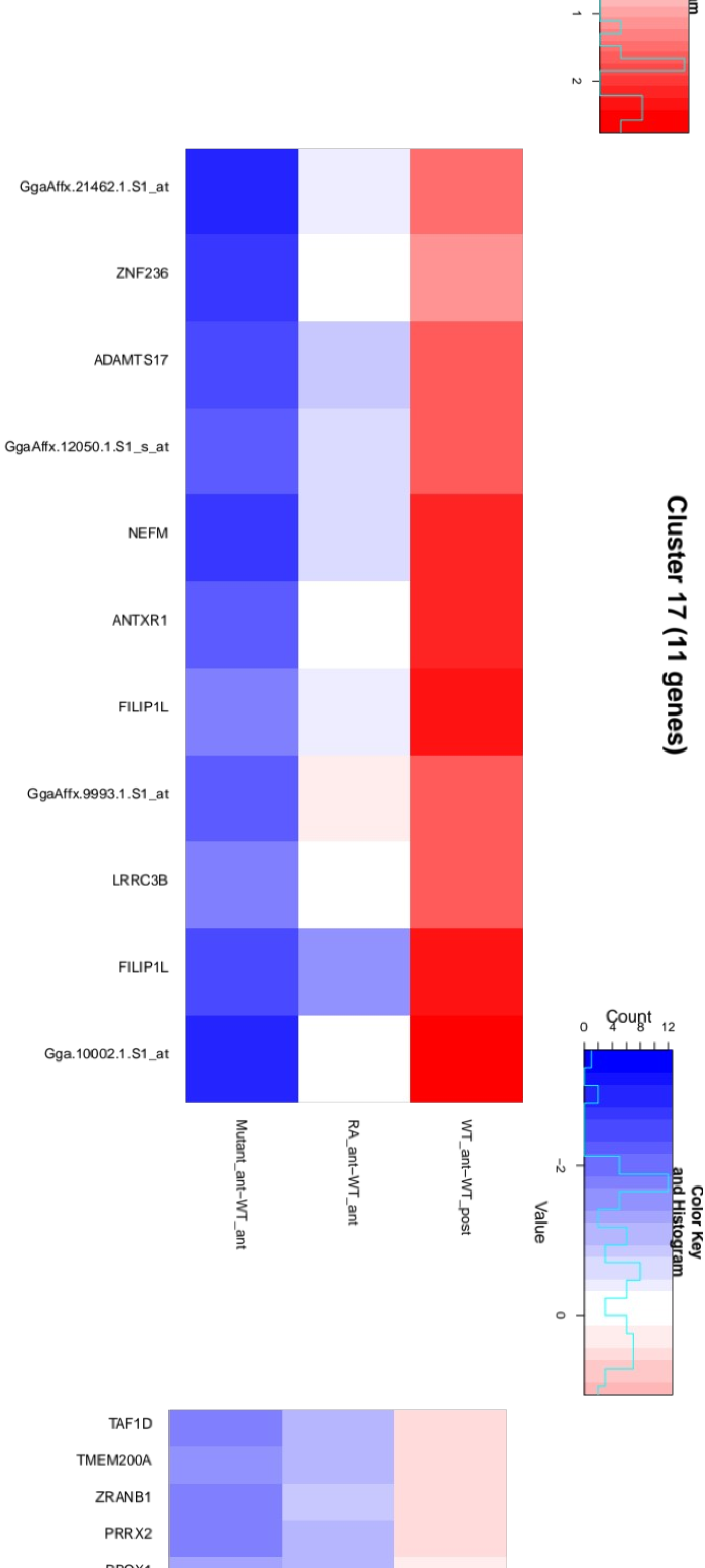

Cluster 17 (11 genes)
GgaAffx.21462.1.S1_at
ZNF236
ADAMTS17
GgaAffx.12050.1.S1_s_at
NEFM
ANTXR1
FILIP1L
GgaAffx.9993.1.S1_at
LRRC3B
FILIP1L
Gga.10002.1.S1_at
Mutant_ant−WT_ant
RA_ant−WT_ant
WT_ant−WT_post
Color Key and Histogram
Count
0 4 8 12
Value
−2
0
1
2
TAF1D
TMEM200A
ZRANB1
PRRX2

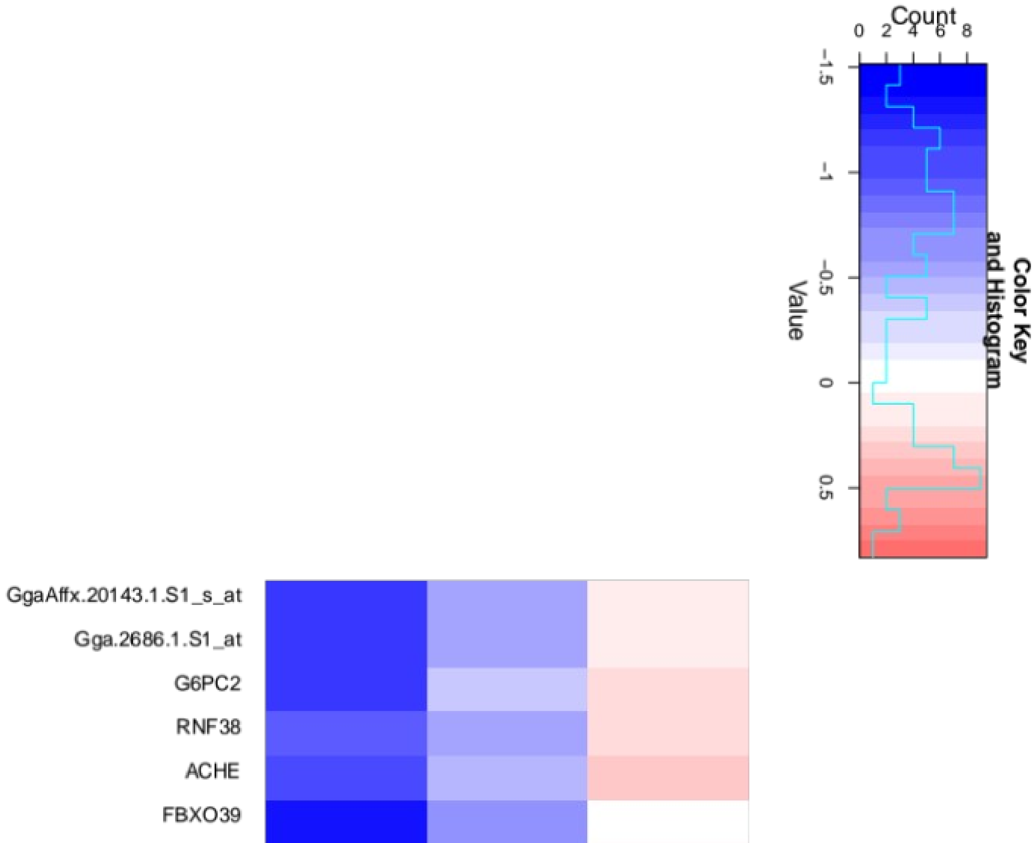
Color Key
and Histogram
Count
0 2 4 6 8
Value
−1.5
−1
−0.5
0
0.5
GgaAffx.20143.1.S1_s_at
Gga.2686.1.S1_at
G6PC2
RNF38
ACHE
FBXO39

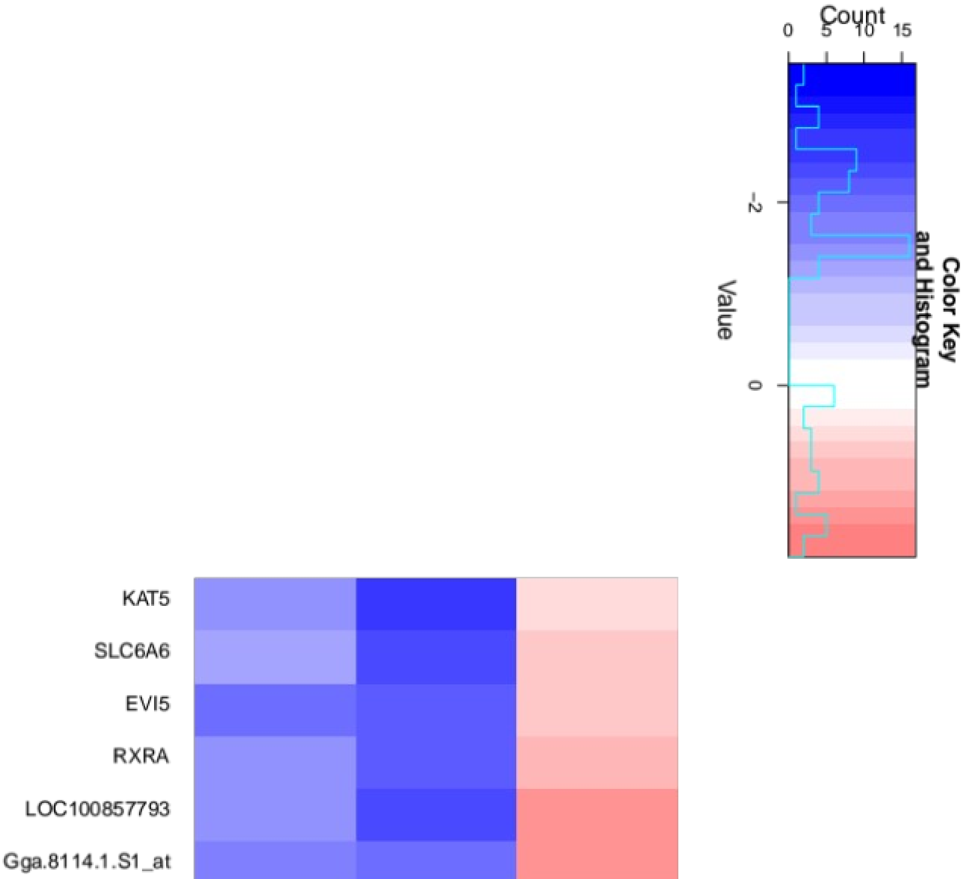
Color Key and Histogram
Count
0
5
10
15
Value
-2
0
KAT5
SLC6A6
EVI5
RXRA
LOC100857793
Gga.8114.1.S1_at

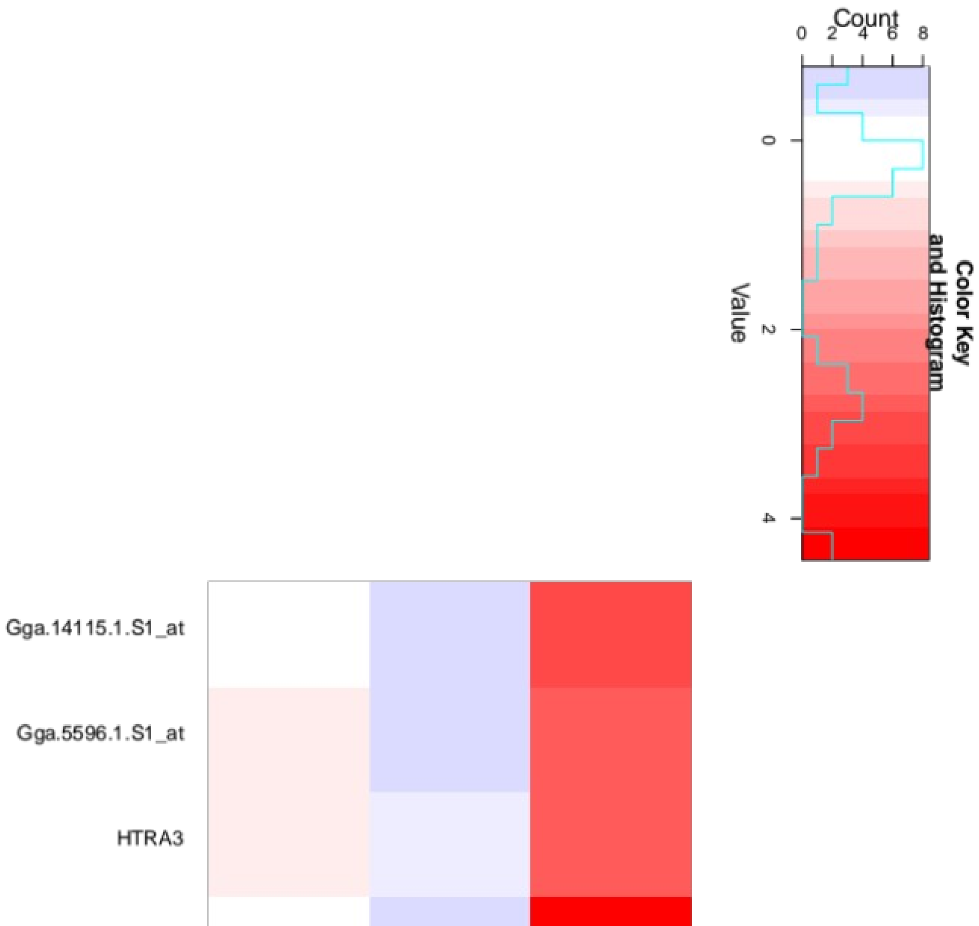
Color Key
and Histogram
Count
0 2 4 6 8
Value
0
2
4
Gga.14115.1.S1_at
Gga.5596.1.S1_at
HTRA3

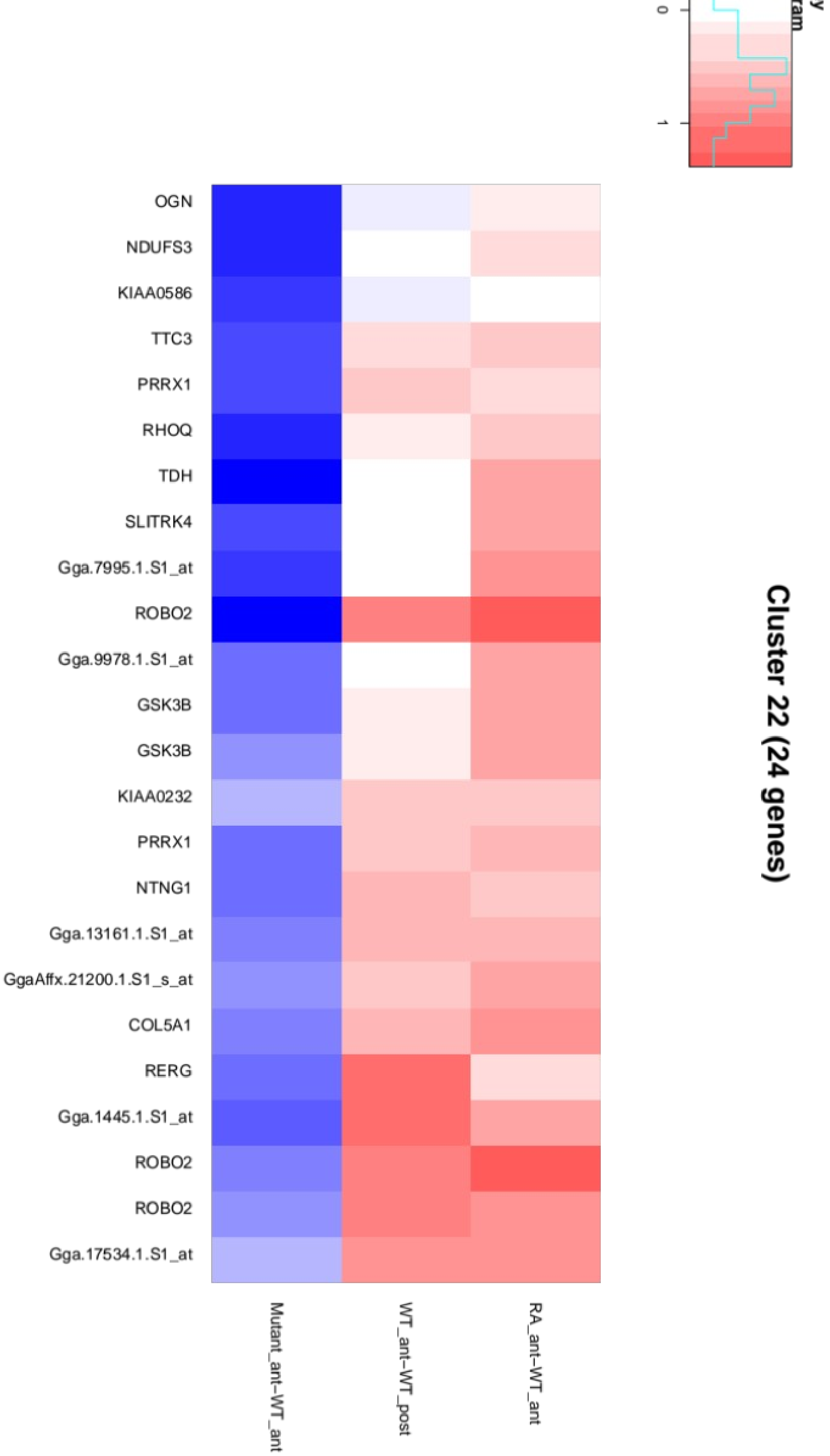
Cluster 22 (24 genes)
OGN
NDUFS3
KIAA0586
TTC3
PRRX1
RHOQ
TDH
SLITRK4
Gga.7995.1.S1_at
ROBO2
Gga.9978.1.S1_at
GSK3B
GSK3B
KIAA0232
PRRX1
NTNG1
Gga.13161.1.S1_at
GgaAffx.21200.1.S1_s_at
COL5A1
RERG
Gga.1445.1.S1_at
ROBO2
ROBO2
Gga.17534.1.S1_at
Mutant_ant−WT_ant
WT_ant−WT_post
RA_ant−WT_ant
0
1

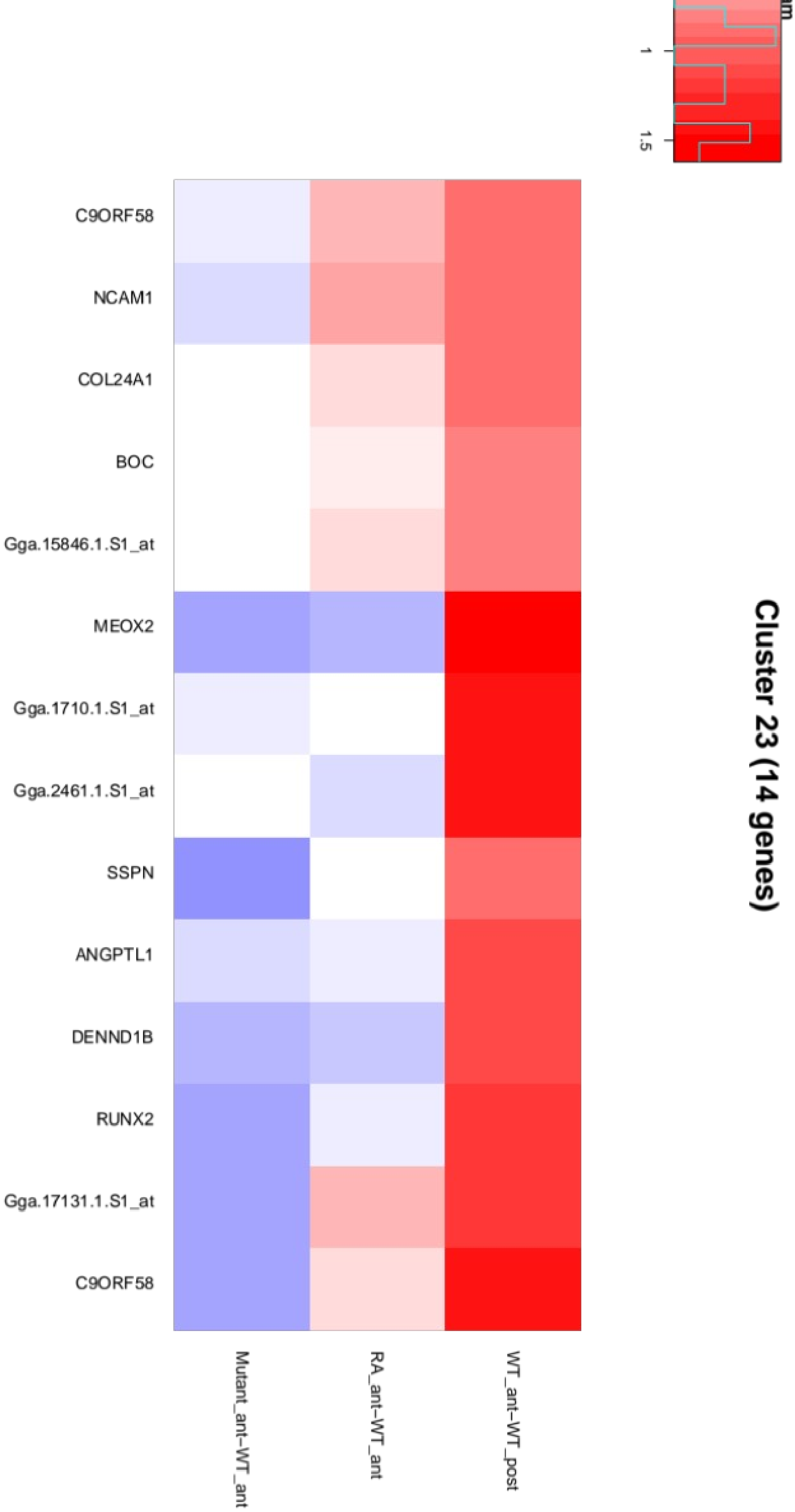
Cluster 23 (14 genes)
C9ORF58
NCAM1
COL24A1
BOC
Gga.15846.1.S1_at
MEOX2
Gga.1710.1.S1_at
Gga.2461.1.S1_at
SSPN
ANGPTL1
DENND1B
RUNX2
Gga.17131.1.S1_at
C9ORF58
Mutant_ant−WT_ant
RA_ant−WT_ant
WT_ant−WT_post
1
1.5

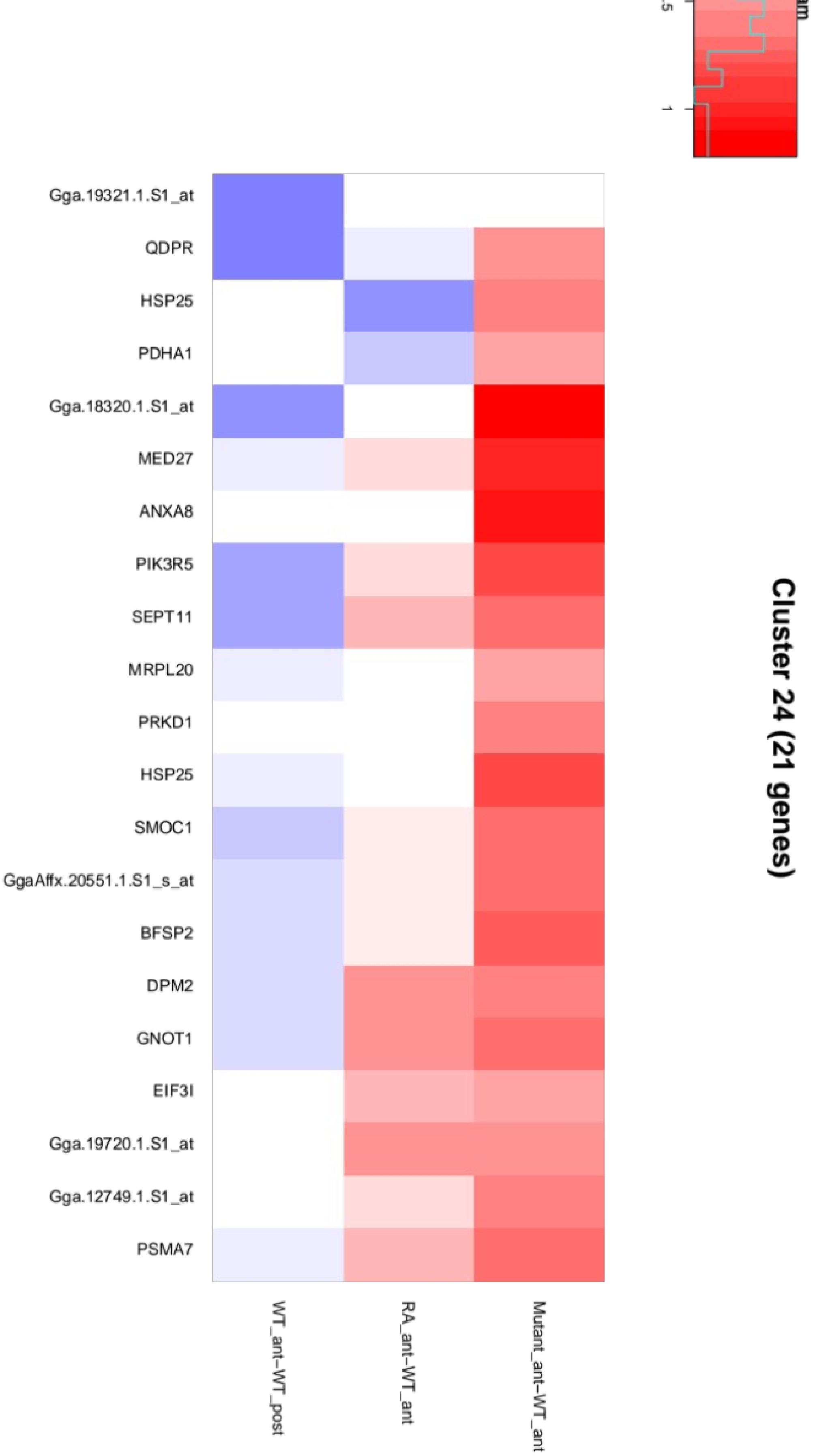
Cluster 24 (21 genes)
Gga.19321.1.S1_at
QDPR
HSP25
PDHA1
Gga.18320.1.S1_at
MED27
ANXA8
PIK3R5
SEPT11
MRPL20
PRKD1
HSP25
SMOC1
GgaAffx.20551.1.S1_s_at
BFSP2
DPM2
GNOT1
EIF3I
Gga.19720.1.S1_at
Gga.12749.1.S1_at
PSMA7
WT_ant−WT_post
RA_ant−WT_ant
Mutant_ant−WT_ant
1

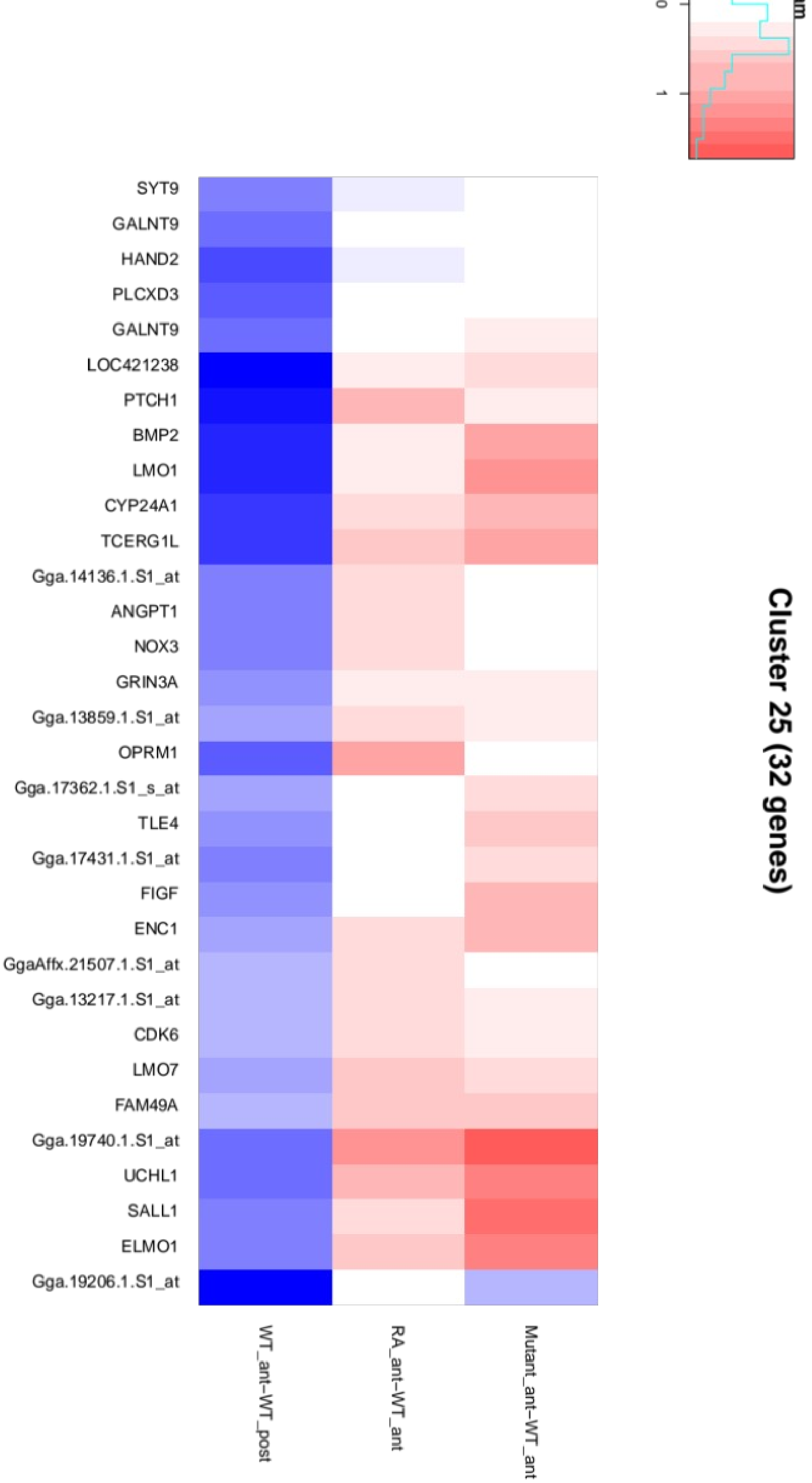
Cluster 25 (32 genes)
SYT9
GALNT9
HAND2
PLCXD3
GALNT9
LOC421238
PTCH1
BMP2
LMO1
CYP24A1
TCERG1L
Gga.14136.1.S1_at
ANGPT1
NOX3
GRIN3A
Gga.13859.1.S1_at
OPRM1
Gga.17362.1.S1_s_at
TLE4
Gga.17431.1.S1_at
FIGF
ENC1
GgaAffx.21507.1.S1_at
Gga.13217.1.S1_at
CDK6
LMO7
FAM49A
Gga.19740.1.S1_at
UCHL1
SALL1
ELMO1
Gga.19206.1.S1_at
WT_ant−WT_post
RA_ant−WT_ant
Mutant_ant−WT_ant
0
1

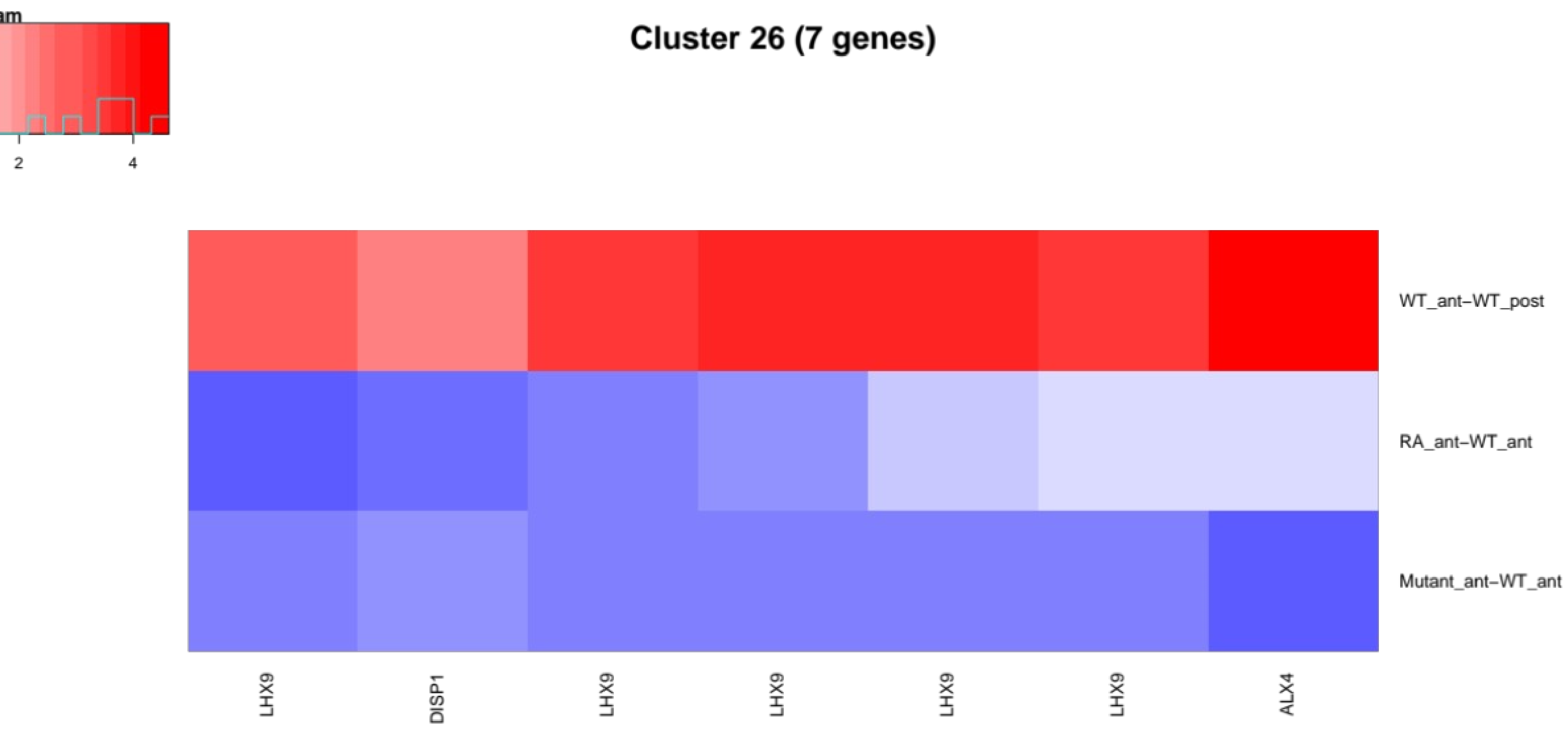
am
2
4
Cluster 26 (7 genes)
WT_ant−WT_post
RA_ant−WT_ant
Mutant_ant−WT_ant
LHX9
DISP1
LHX9
LHX9
LHX9
LHX9
ALX4

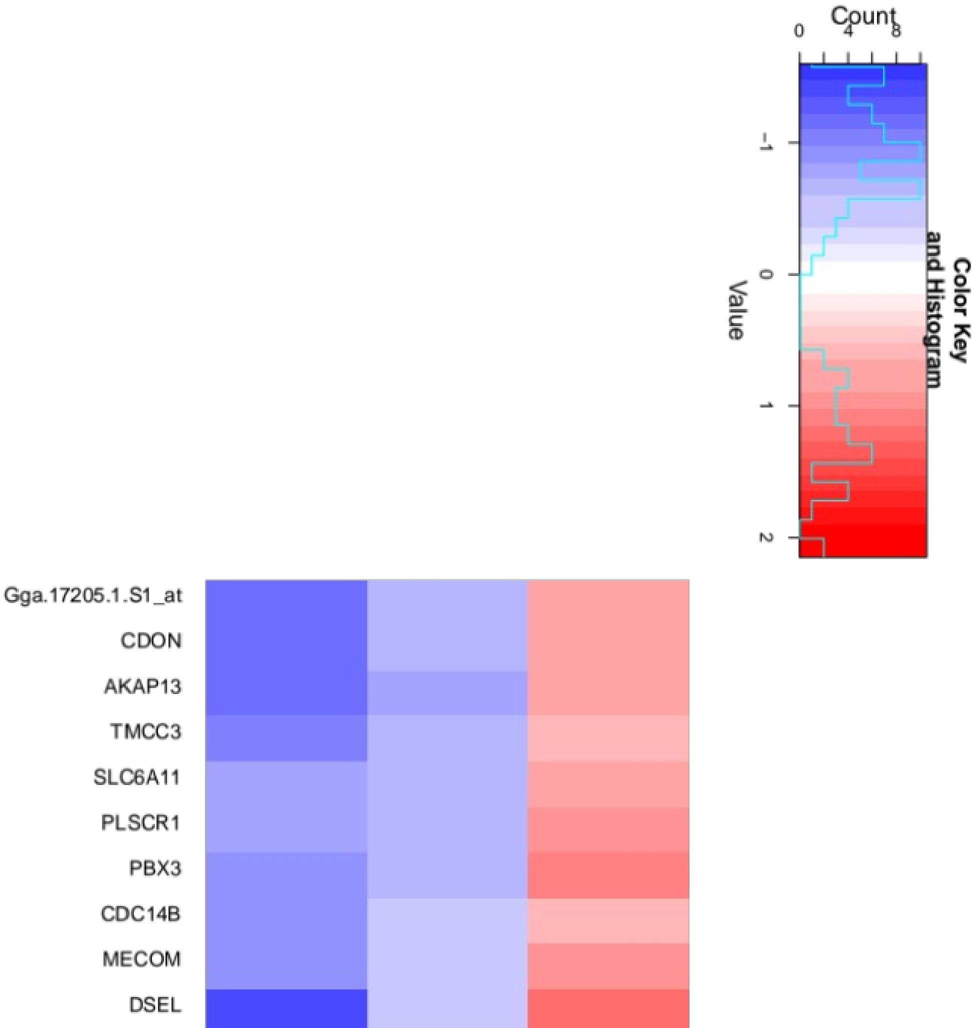

Color Key
and Histogram
Count
0
4
8
Value
-1
0
1
2
Gga.17205.1.S1_at
CDON
AKAP13
TMCC3
SLC6A11
PLSCR1
PBX3
CDC14B
MECOM
DSEL

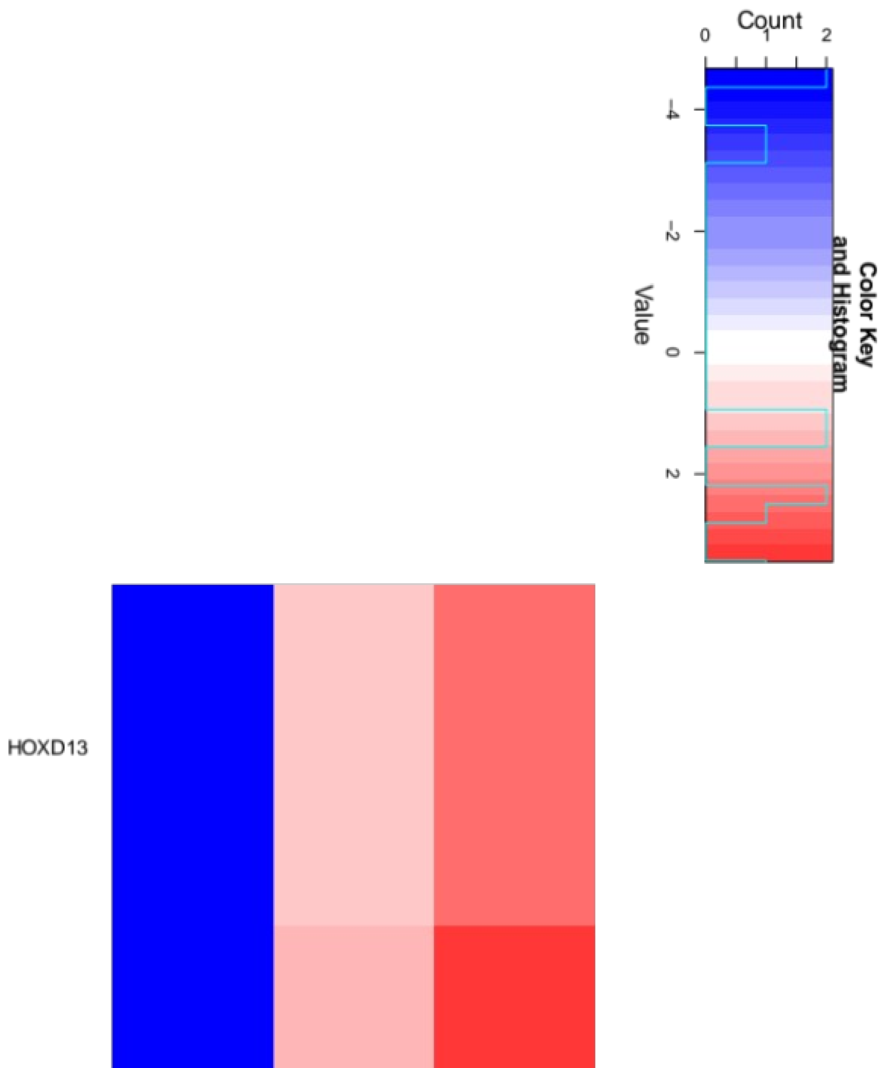
Color Key
and Histogram
Count
0
1
2
Value
−4
−2
0
2
HOXD13

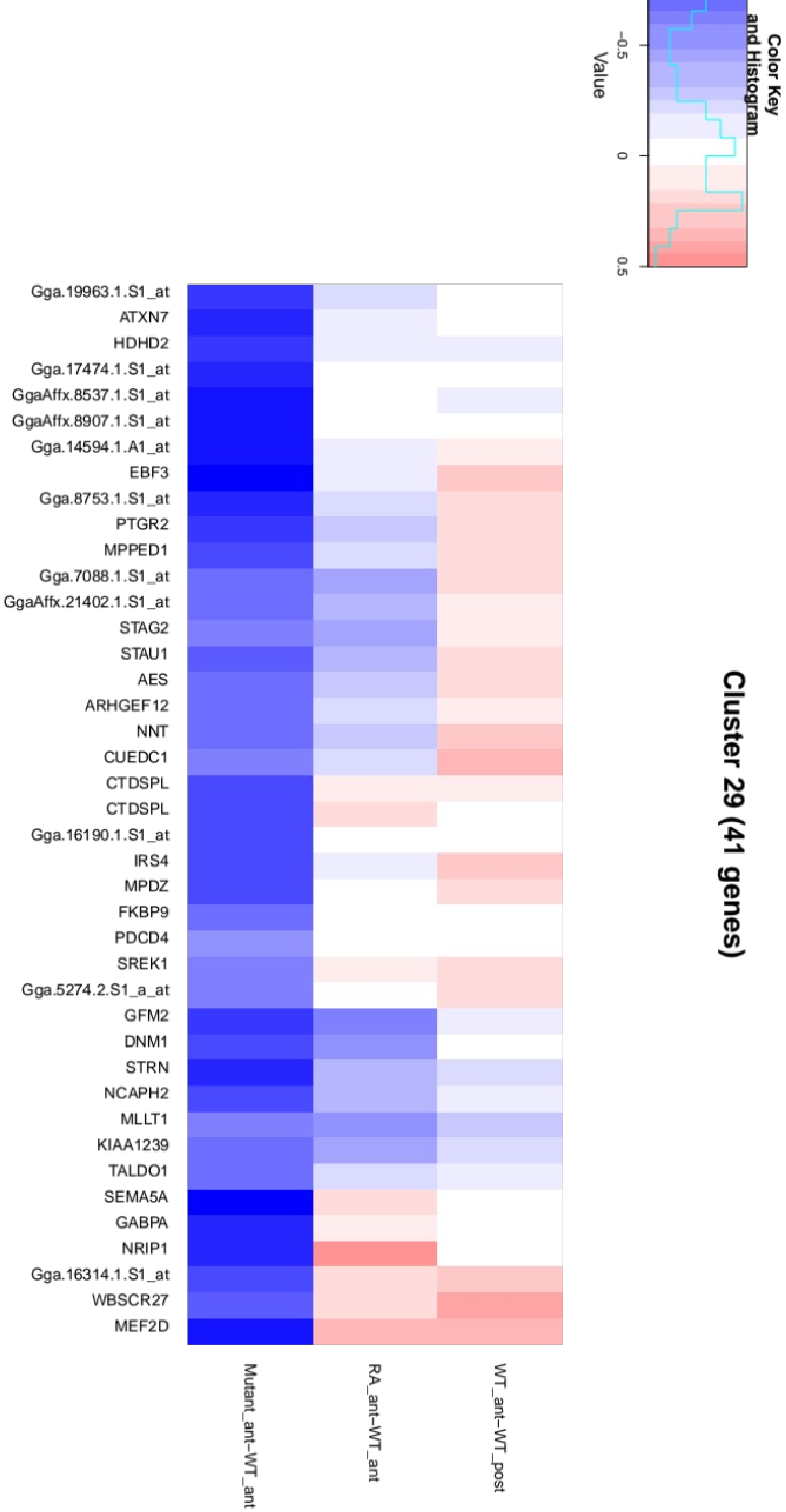

Color Key and Histogram
Value
−0.5
0
0.5
Cluster 29 (41 genes)
Gga.19963.1.S1_at
ATXN7
HDHD2
Gga.17474.1.S1_at
GgaAffx.8537.1.S1_at
GgaAffx.8907.1.S1_at
Gga.14594.1.A1_at
EBF3
Gga.8753.1.S1_at
PTGR2
MPPED1
Gga.7088.1.S1_at
GgaAffx.21402.1.S1_at
STAG2
STAU1
AES
ARHGEF12
NNT
CUEDC1
CTDSPL
CTDSPL
Gga.16190.1.S1_at
IRS4
MPDZ
FKBP9
PDCD4
SREK1
Gga.5274.2.S1_a_at
GFM2
DNM1
STRN
NCAPH2
MLLT1
KIAA1239
TALDO1
SEMA5A
GABPA
NRIP1
Gga.16314.1.S1_at
WBSCR27
MEF2D
Mutant_ant−WT_ant
RA_ant−WT_ant
WT_ant−WT_post
